# Histone H3 lysine 4 methylation recruits DNA demethylases to enforce gene expression in *Arabidopsis*

**DOI:** 10.1038/s41477-025-01924-y

**Published:** 2025-02-11

**Authors:** Ming Wang, Yan He, Zhenhui Zhong, Ashot Papikian, Shuya Wang, Jason Gardiner, Basudev Ghoshal, Suhua Feng, Yasaman Jami-Alahmadi, James A. Wohlschlegel, Steven E. Jacobsen

**Affiliations:** 1 State Key Laboratory of Integrated Management of Pest Insects and Rodents, Institute of Zoology, Chinese Academy of Sciences, Beijing, China; 2 Department of Molecular, Cell and Developmental Biology, University of California Los Angeles, Los Angeles, CA USA; 3 Eli and Edyth Broad Center of Regenerative Medicine and Stem Cell Research, University of California Los Angeles, Los Angeles, CA USA; 4 Department of Biological Chemistry, University of California Los Angeles, Los Angeles, CA USA; 5 Howard Hughes Medical Institute (HHMI), UCLA, Los Angeles, CA USA; 6 Present Address: Ministry of Education Key Laboratory for Bio-Resource and Eco-Environment, College of Life Sciences, State Key Laboratory of Hydraulics and Mountain River Engineering, Sichuan University, Chengdu, China; 7 Present Address: Plant Molecular and Cellular Biology Laboratory, Salk Institute for Biological Studies, La Jolla, CA USA; 8 Present Address: Translational Plant Biology, Department of Biology, Utrecht University, Utrecht, The Netherlands; 9 Present Address: Summerland Research and Development Centre, Agriculture and Agri-Food Canada, Summerland, British Columbia Canada

**Keywords:** Plant molecular biology, Epigenetics

## Abstract

Patterning of DNA methylation in eukaryotic genomes is controlled by de novo methylation, maintenance mechanisms and demethylation pathways. In *Arabidopsis thaliana*, DNA demethylation enzymes are clearly important for shaping methylation patterns, but how they are regulated is poorly understood. Here we show that the targeting of histone H3 lysine four trimethylation (H3K4me3) with the catalytic domain of the SDG2 histone methyltransferase potently erased DNA methylation and gene silencing at *FWA* and also erased CG DNA methylation in many other regions of the *Arabidopsis* genome. This methylation erasure was completely blocked in the *ros1 dml2 dml3* triple mutant lacking DNA demethylation enzymes, showing that H3K4me3 promotes the active removal of DNA methylation. Conversely, we found that the targeted removal of H3K4me3 increased the efficiency of targeted DNA methylation. These results highlight H3K4me3 as a potent anti-DNA methylation mark and also pave the way for development of more powerful epigenome engineering tools.

## Abstract (web summary)

This study revealed that targeting H3K4me3 via the H3K4 methyltransferase SDG2 activates gene expression and removes DNA methylation by recruiting DNA demethylases. Conversely, the removal of H3K4me3 synergistically enhances targeted DNA methylation.

## Main

DNA methylation is a conserved epigenetic mark usually associated with gene silencing^1,2^. In plants, DNA methylation patterns are often stably inherited between sexual generations, and many epigenetic alleles with important phenotypes have been described that have identical DNA sequences but differ in their methylation and expression states^3,4^. DNA methylation in plants is established by the RNA-directed DNA methylation (RdDM) pathway in all sequence contexts (CG, CHG (H = A, T, C) and CHH) and is maintained by different DNA methyltransferase systems^2,5–7^. Although cytosines in all sequence contexts can be methylated in plant genomes^8^, DNA methylation in the CG sequence context is usually the critical type for the maintenance of gene silencing^2,3,9,10^. Loss of DNA methylation can occur passively upon replication in the absence of functional maintenance by DNA methyltransferases. Alternatively, active demethylation in plants involves a family of glycosylases including REPRESSOR OF SILENCING 1 (ROS1), DEMETER (DME), DEMETER-LIKE 2 (DML2) and DML3^11–13^. ROS1, DML2 and DML3 function in vegetative tissues, while DME mostly functions in reproductive tissues^13^.

In contrast to sites of gene silencing marked by DNA methylation, sites of active chromatin in plants are associated with various positive epigenetic marks including trimethylation of H3K4. A number of histone methyltransferases in *Arabidopsis* control methylation at the H3K4 position, and SET DOMAIN PROTEIN 2 (SDG2) appears to be one of the major enzymes depositing histone H3 lysine four trimethylation (H3K4me3)^14,15^. The removal of H3K4 methylation is controlled by H3K4 demethylases, which include the Jumonji domain containing protein JMJ14-18^16–19^, as well as LYSINE SPECIFIC DEMETHYLASE LIKE (LDL1-3) and FLOWERING LOCUS D (FLD)^20–22^.

We previously found that targeting the Sss1/MQ1 bacterial CG-specific DNA methyltransferase to the *FLOWERING WAGENINGEN* (*FWA*) gene with deactivated CRISPR/Cas9 (dCas9) caused the establishment of DNA methylation and gene silencing^23^. SssI was able to directly install CG methylation in a manner that did not require the RdDM pathway normally responsible for the establishment of DNA methylation in plants^23^. We also found that *Arabidopsis* plants highly expressing SssI showed widespread ectopic CG DNA methylation throughout the genome^24^. However, many genomic regions were refractory to methylation establishment, most notably promoters of protein coding genes containing positive epigenetic marks such as histone acetylation and H3K4me3^24,25^. These results were consistent with the general lack of DNA methylation in promoters of expressed genes^26–28^ and raised the possibility that one or more positive epigenetic marks might actively resist DNA methylation. In this article, we demonstrate that the targeting of H3K4me3 to specific loci actively erases DNA methylation by recruiting DNA demethylases. We also show that targeting H3K4me3 demethylation greatly facilitates the establishment of DNA methylation and gene silencing. These results facilitate our understanding of epigenetic pathways in plants and outline important principals for efficient chromatin engineering.

## Results

### Targeting SDG2 catalytic domain erases CG DNA methylation

**Fig. 1:**
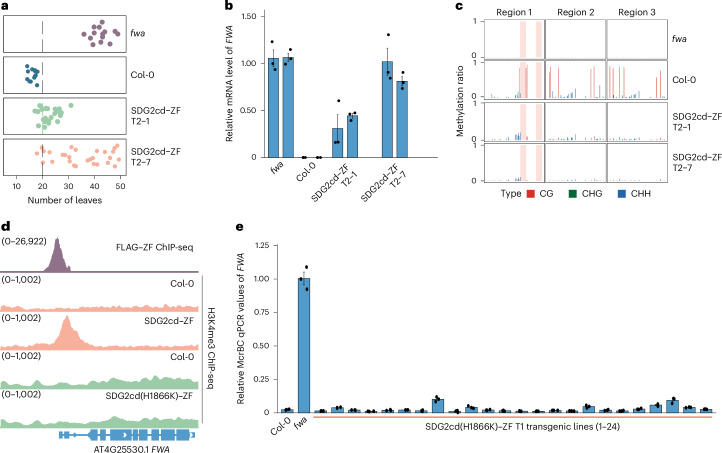
Targeting SDG2cd using ZF triggered gene activation by H3K4me3 deposition. **a**, Dot plots representing the leaf number of *fwa*, Col-0 (WT) and two T2 lines of SDG2cd–ZF. **b**, Quantitative reverse transcription PCR (qRT–PCR) indicating the relative messenger RNA levels of *FWA* in *fwa*, Col-0 and two T2 transgenic lines of SDG2cd–ZF (*n* = 4 biological replicates). **c**, BS-PCR-seq showing CG, CHG and CHH DNA methylation levels at *FWA* promoter regions in *fwa*, Col-0 and two T2 transgenic lines of SDG2cd–ZF. Pink vertical boxes indicate ZF binding sites. **d**, Genome browser view depicting FLAG–ZF ChIP-seq, and H3K4me3 ChIP-seq signals in either Col-0, SDG2cd–ZF or SDG2cd(H1866K)–ZF. The numbers in parentheses indicate the data range of the ChIP-seq signals (RPKM, reads per kilobase million). **e**, Relative McrBc-qPCR values in Col-0, *fwa* and T1 transgenic lines of SDG2cd(H1866K)–ZF site mutation in the Col-0 background (*n* = 24 biological replicates). A lower value indicates a relatively higher level of DNA methylation. Data are presented as mean values ± s.e.m. in **b** and **e**.

**Extended Data Fig. 1:**
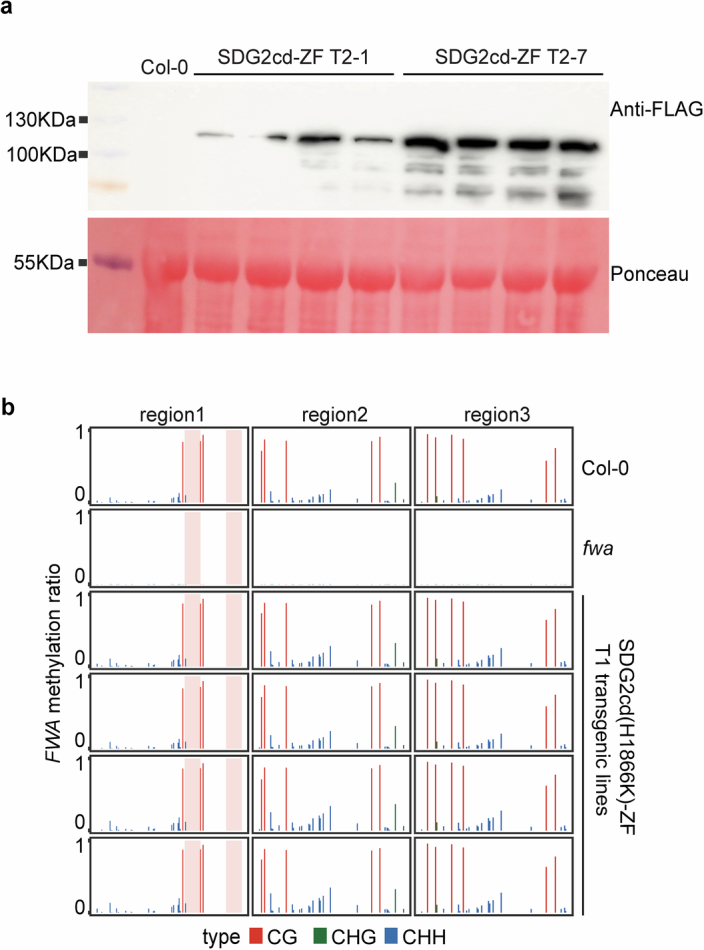
The catalytic activity of SDG2 is required for the removal of DNA methylation by SDG2cd-ZF. **a**, Western blotting showing the protein expression level of SDG2cd-ZF in two representative T2 transgenic lines. **b**, BS-PCR-seq showing CG, CHG and CHH DNA methylation levels at *FWA* promoter regions in Col-0, *fwa* and four representative T1 transgenic lines of SDG2cd(H1866K)-ZF in the Col-0 background. Source data

The previous findings of an anticorrelation of H3K4me3 and CG DNA methylation^24,27,28^ prompted us to directly test whether the establishment of H3K4me3 might antagonize CG methylation. *SDG2* encodes the major H3K4me3 methyltransferase in *Arabidopsis*^14,15^. To test whether SDG2 could be used for H3K4me3 targeting, we fused the catalytic domain of SDG2 (SDG2cd) with the artificial zinc finger ZF108 (SDG2cd–ZF), which was designed to target the *Arabidopsis* *FWA* gene^25,29^. *FWA* serves as a valuable endogenous reporter gene because it is silenced in the wild-type Col-0 background by dense CG DNA methylation in its promoter region, while in *fwa* epialleles, this DNA methylation has been heritably lost, resulting in *FWA* overexpression and an easy to score late-flowering phenotype^3,30,31^. An SDG2cd–ZF fusion driven by the *UBQ10* promoter was transformed into wild-type plants to test whether it could remove *FWA* DNA methylation and cause activation of *FWA* expression. Indeed, we found that many SDG2cd–ZF transgenic lines showed a late-flowering phenotype accompanied by the activation of *FWA* expression and an almost complete removal of DNA methylation as measured by bisulfite amplicon sequencing (BS-PCR-seq) (Fig. 1a–c). The variation of *FWA* activation, removal of DNA methylation and the late-flowering phenotype across different transgenic lines was notably correlated with the protein expression level of SDG2cd–ZF (Fig. 1a–c and Extended Data Fig. 1a). To validate that the loss of *FWA* DNA methylation was triggered by SDG2-mediated deposition of H3K4me3, we performed H3K4me3 chromatin immunoprecipitation followed by sequencing (ChIP-seq). As expected, we observed a robust peak of H3K4me3 at the *FWA* promoter in the SDG2cd–ZF plants (Fig. 1d). These results suggest that the targeting of H3K4me3 to *FWA* can powerfully reduce DNA methylation and induce *FWA* expression.

To further confirm that the H3K4me3 deposition and removal of DNA methylation are dependent on the catalytic activity of SDG2, we generated an H1866K missense mutation version of SDG2cd–ZF based on a similar mutation reported in the yeast H3K4 methyltransferase^32,33^. It is worth noting that the H1866K mutation of SDG2cd–ZF completely blocked H3K4me3 deposition and DNA methylation removal at the *FWA* locus (Fig. 1d,e and Extended Data Fig. 1b), indicating that the catalytic activity of SDG2 is required for DNA demethylation.

**Fig. 2:**
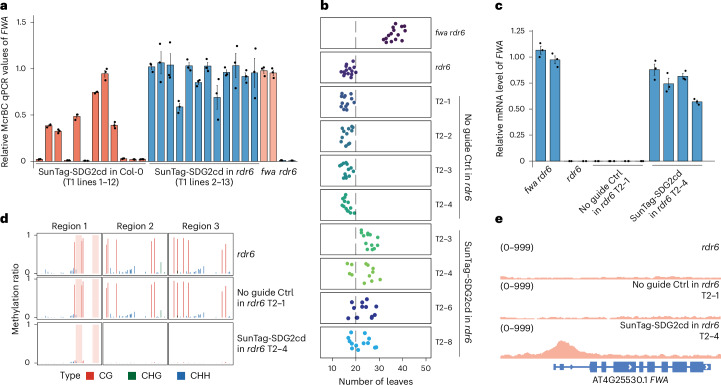
SunTag–SDG2cd also triggered gene activation and H3K4me3 deposition. **a**, Relative McrBC-qPCR values for the *fwa*, *rdr6* and T1 transgenic lines of SunTag–SDG2cd in both the Col-0 and *rdr6* backgrounds, respectively (*n* = 12 biological replicates). A lower value indicates a relatively higher level of DNA methylation. **b**,**c**, Dot plots indicating the leaf number (**b**) and qRT–PCR assays showing the relative mRNA levels of *FWA* (**c**), in *fwa rdr6*, *rdr6*, and T2 transgenic lines of SunTag–SDG2cd with no guide control (Ctrl) and SunTag–SDG2cd targeting the *FWA* gene in the *rdr6* background (*n* = 4 biological replicates). **d**,**e**, Bisulfite PCR-seq results depicting the relative CG, CHG and CHH DNA methylation levels of the *FWA* promoter (**d**) and genome browser view showing H3K4me3 ChIP-seq signals at the *FWA* locus (**e**), in *rdr6* and T2 transgenic lines of SunTag–SDG2cd with no guide or SunTag–SDG2cd targeting the *FWA* gene in the *rdr6* background. The numbers in parentheses indicate the data range of the ChIP-seq signals (RPKM). Data are presented as mean values ± s.e.m. in **a** and **c**.

**Extended Data Fig. 2:**
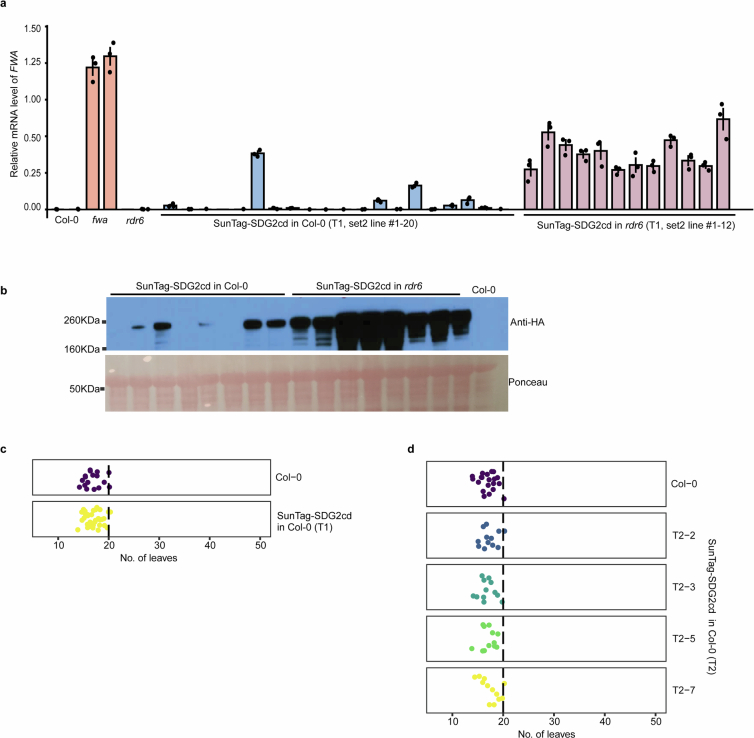
SunTag-SDG2cd exhibited higher *FWA* mRNA levels and dCas9 protein expression level in the *rdr6* background compared to the Col-0 background. **a**, Histogram showing the relative mRNA level of *FWA* in Col-0, *fwa*, *rdr6*, as well as T1 transgenic lines of SunTag-SDG2cd in Col-0 (n = 20 biological replicates) and *rdr6* (n = 12 biological replicates) background, respectively. Data are presented as mean values ± s.e.m. **b**, Western blotting results representing the protein expression levels of HA-dCas9 in both the Col-0 background and T1 transgenic lines of SunTag-SDG2cd in the Col-0 and *rdr6* mutant backgrounds, respectively. **c-d**, The dot plots showing the leaf numbers of Col-0 and SunTag-SDG2cd transgenic lines in the Col-0 background at T1 (**c**) and T2 (**d**) generations, respectively. Source data

SDG2cd was also cloned into the dCas9-based SunTag system (SunTag–SDG2cd) together with guide RNAs directed to the *FWA* promoter^30,34^. In this system, dCas9 is fused to 10 repeats of the GCN4 peptide, and separately a single-chain antibody that recognizes these repeats is fused to GFP and SDG2cd. We found that while SunTag–SDG2cd activated some *FWA* expression and removal of DNA methylation in the Col-0 background (Fig. 2a and Extended Data Fig. 2a,b), it failed to induce a late-flowering phenotype in either first transgenic (T1) or T2 generations that we examined (Extended Data Fig. 2c,d). We previously found that dCas9 expression levels were much higher when introduced into the *rdr6* background that reduces transgene silencing^31^. We therefore also transformed SunTag–SDG2cd into *rdr6*, where we found that dCas9 expression was markedly increased (Extended Data Fig. 2b). As a result, the activation of *FWA* and removal of DNA methylation by SunTag–SDG2cd in *rdr6* T1 lines was much higher (Fig. 2a and Extended Data Fig. 2a), and some of the T2 lines showed an intermediate late-flowering phenotype (Fig. 2b,c). These plants also showed an almost complete loss of *FWA* DNA methylation (Fig. 2d) and a robust peak of H3K4me3 at the *FWA* promoter (Fig. 2e). Together, these results suggest that H3K4me3 targeting antagonizes DNA methylation.

### SDG2cd–ZF-mediated DNA demethylation across diverse regions

**Fig. 3:**
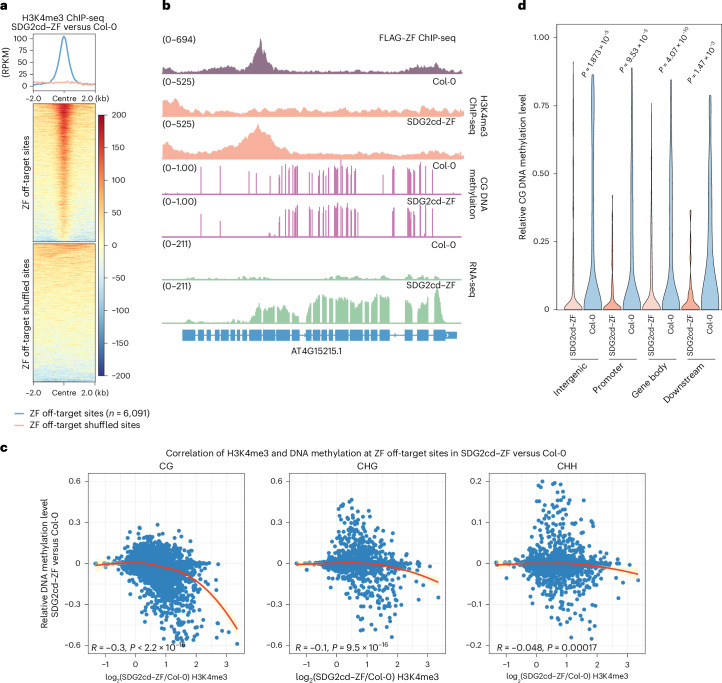
Targeting SDG2cd erased DNA methylation. **a**, Metaplot and heat maps showing H3K4me3 ChIP-seq signals of SDG2cd–ZF versus Col-0 over ZF off-target and shuffled sites (*n* = 6,091), respectively. The colors and values on the right side of heat map indicates the scale bar of the heat map. **b**, Genome browser view showing the H3K4me3 ChIP-seq signals, CG DNA methylation levels and RNA-seq signals in Col-0 and SDG2cd–ZF at an example of ZF off-target gene. FLAG–ZF ChIP-seq signal indicates the ZF binding site, and the numbers in parentheses indicate the data range of the ChIP-seq signals (RPKM). **c**, Scatterplots showing the correlation of CG, CHG and CHH DNA methylation levels with log_2_ H3K4me3 ChIP-seq signals in SDG2cd–ZF versus Col-0 over ZF off-target sites (*n* = 6,091). Statistical tests were two-sided, and *P* values were reported without adjustment for multiple comparisons. **d**, Violin plot representing the relative CG DNA methylation levels of Col-0 and SDG2cd–ZF across ZF binding genes with strong increased H3K4me3, spanning the intergenic, promoter, gene body and downstream regions of genes. The *P* values were calculated using two-sided *t*-test.

It is known that ZF108 (ZF) binds not only to *FWA* but also to thousands of off-target sites throughout the *Arabidopsis* genome^25,31^, which allowed us to examine the effects of targeting H3K4me3 to many other sites. Analysis of ChIP-seq data showed a substantial accumulation of H3K4me3 in SDG2cd–ZF plants at the majority of the 6,091 sites with a robust ZF ChIP-seq peak (Fig. 3a,b).

**Extended Data Fig. 3:**
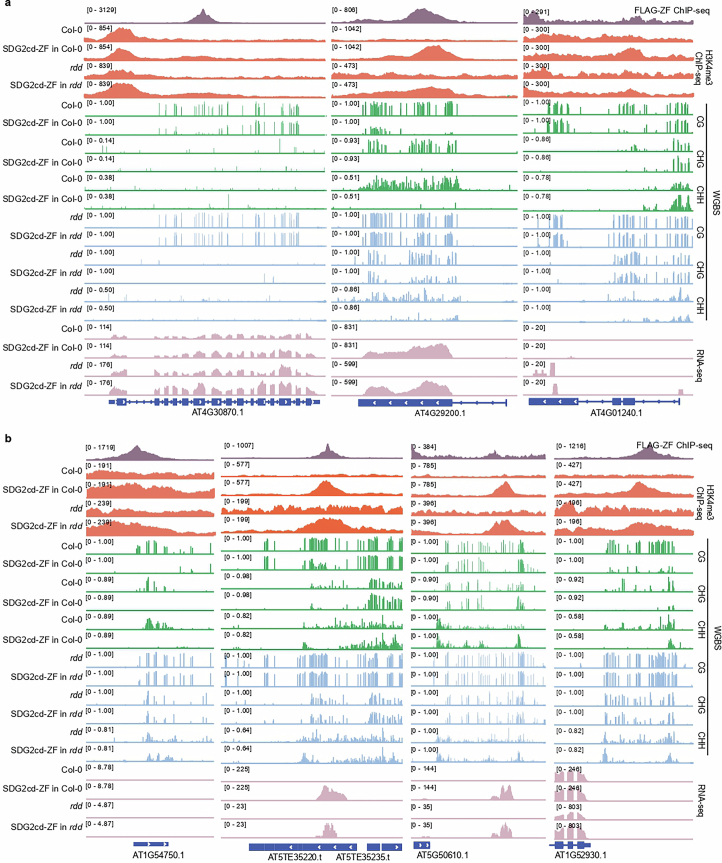
SDG2cd-ZF reduced DNA methylation at ZF off-target sites in the Col-0 wild type background but not in the *rdd* mutant background. **a-b** Genome browser views showing H3K4me3 ChIP-seq signals, CG, CHG, and CHH DNA methylation levels, and RNA-seq signals at gene body regions (**a**), TEs (**b**, left two panels), and intergenic regions (**b**, right two panels) in Col-0, SDG2cd-ZF in the Col-0 background, *rdd*, and SDG2cd-ZF in the *rdd* mutant background. FLAG-ZF ChIP-seq signals indicate the ZF binding sites. The numbers in the parentheses indicate the data range of the ChIP-seq signals (RPKM).

We compared the methylation profiles of SDG2cd–ZF and wild-type plants using whole-genome bisulfite sequencing (WGBS) and found an inverse correlation between the levels of H3K4me3 and DNA methylation at these ZF off-target sites, especially for CG methylation (Fig. 3b,c). Most ZF binding sites (5,212 out of 6,091, or 85%) showed increased H3K4me3 in SDG2cd–ZF (Supplementary Table 1). Among them, 1,287 out of 5,212 ZF binding sites had pre-existing DNA methylation levels in Col-0 (using 20% CG methylation as a threshold), and around 65% of these (834 out of 1,287) showed lower DNA methylation levels in SDG2cd–ZF compared with Col-0. These results suggest that induced H3K4me3 antagonizes CG methylation broadly throughout the genome. Around one third of *Arabidopsis* genes contain CG methylation in their transcribed regions (gene body methylation). In cases where the ZF peak corresponded to a region of gene body methylation, there was a consistent loss of methylation in a focal region corresponding to the H3K4me3 peaks introduced by SDG2cd–ZF (Fig. 3b,d, Extended Data Fig. 3a and Supplementary Fig. 1), showing that H3K4me3 targeting erases gene body methylation. In addition, we occasionally observed ectopic intragenic transcription initiating at H3K4me3 peaks that extended toward the 3′ end of the normally transcribed region (Fig. 3b and Extended Data Fig. 3a), but there were also many examples where there was no change in gene expression (Extended Data Fig. 3a, right panel). This result suggests that H3K4me3 is not always sufficient to drive expression and that expression changes are unlikely to be driving the loss of DNA methylation. In cases where the ZF peak corresponded to transposable elements or DNA methylated intergenic regions, the peak of H3K4me3 was associated with the removal of CG, CHG and CHH DNA methylation and often, but not always, associated with increased expression (Extended Data Fig. 3b). These data suggest that the antagonism between H3K4me3 and DNA methylation is not limited to normal transcriptional start sites (TSSs) but also occurs at many sites in the genome including sites of gene body CG DNA methylation and transposable elements.

### H3K4me3 targeting recruits DNA demethylases

**Fig. 4:**
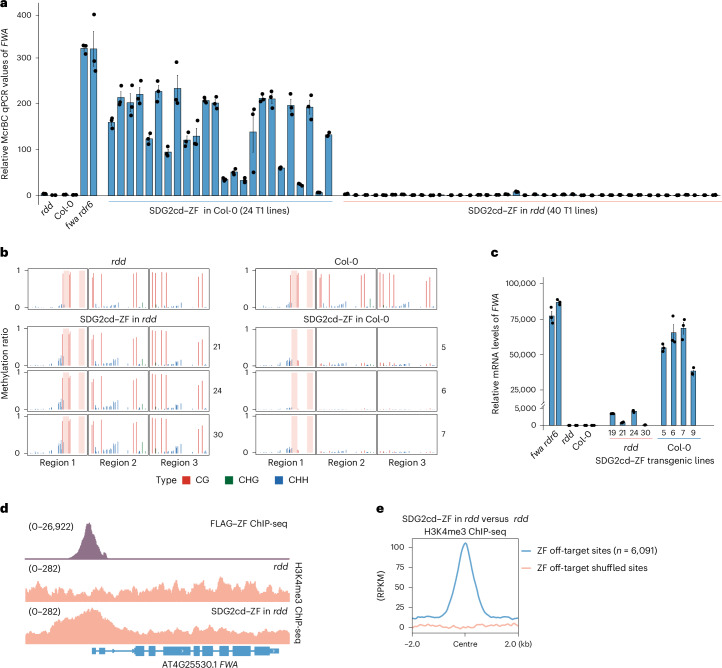
SDG2cd–ZF-mediated DNA demethylation is dependent on DNA demethylases. **a**, Relative McrBc-qPCR values of *FWA in rdd*, Col-0, *fwa rdr6* and SDG2cd–ZF transgenic lines in the Col-0 (*n* = 24 biological replicates) or *rdd* mutant (*n* = 40 biological replicates) backgrounds. A lower value indicates a relatively higher level of DNA methylation. **b**, BS-PCR-seq showing CG, CHG and CHH DNA methylation levels at *FWA* promoter regions in *rdd*, Col-0 and three T1 transgenic lines of SDG2cd–ZF in the *rdd* mutant background (left panel) and Col-0 background (right panel). Pink vertical boxes indicate the ZF binding sites. **c**, qRT–PCR results indicating the relative mRNA levels of *FWA* in *fwa rdr6*, *rdd*, Col-0 and four representative SDG2cd–ZF T1 transgenic lines in the *rdd* mutant or Col-0 backgrounds (*n* = 4 biological replicates). **d**, Genome browser view showing H3K4me3 ChIP-seq signals at the *FWA* region in the *rdd* mutant and SDG2cd–ZF transgenic lines in the *rdd* mutant background. FLAG–ZF ChIP-seq indicates the ZF binding site, and the numbers in parentheses indicate the data range of the ChIP-seq signals (RPKM). **e**, A metaplot showing the normalized H3K4me3 ChIP-seq signals in the SDG2cd–ZF transgenic lines in the *rdd* mutant background versus the *rdd* mutant at ZF off-target and shuffled sites (*n* = 6,091), respectively. Data are presented as mean values ± s.e.m. in **a** and **c**.

**Extended Data Fig. 4:**
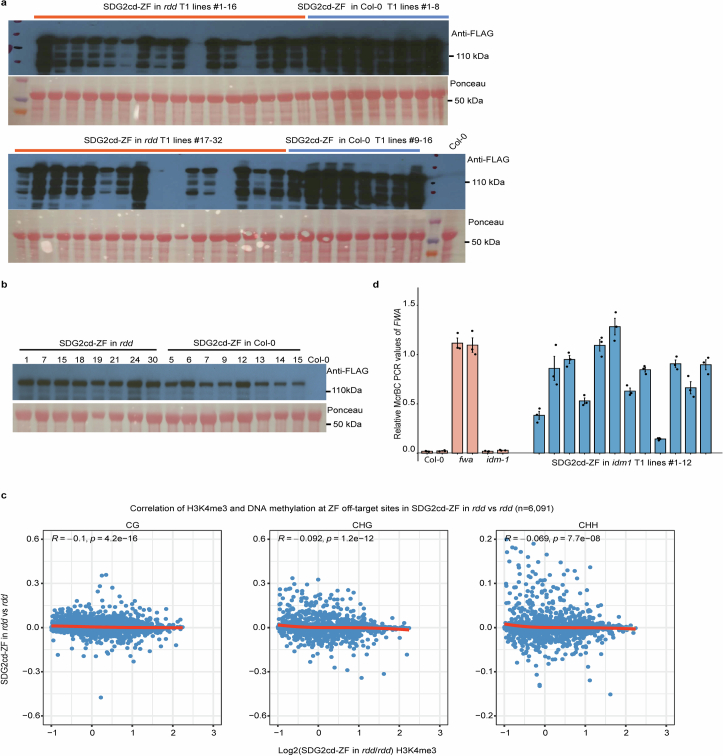
The removal of DNA methylation by SDG2cd-ZF is blocked in the *rdd* mutant but not in the *idm1* mutant, and this effect is not due to the inhibition of protein expression. **a-b** The western blotting results showing the protein expression levels of SDG2cd-ZF in the *rdd* mutant and Col-0 background. The ponceau staining indicates the loading control for each sample. **c**, Scatterplots depicting the correlation of DNA methylation levels and Log2 H3K4me3 ChIP-seq signals in SDG2cd-ZF transgenic lines in the *rdd* mutant background versus *rdd* mutant at ZF off-target sites (n = 6,091). Statistical tests were two-sided, and *p*-values were reported without adjustment for multiple comparisons. **d**, Histogram depicting the relative McrBc-qPCR values of *FWA* in Col-0, *fwa*, *idm1* mutant, and T1 transgenic lines of SDG2cd-ZF in the *idm1* mutant background (n = 12 biological replicates), respectively. A lower value indicates a relatively higher level of DNA methylation. Data are presented as mean values ± s.e.m. Source data

We next sought to investigate the mechanism by which H3K4me3 gain results in the loss of CG DNA methylation. Theoretically, the loss could be either due to a failure of CG methylation maintenance or caused by the active removal of DNA methylation by DNA demethylases. *Arabidopsis* expresses three DNA demethylases in adult plant tissues called *ROS1*, *DML2* and *DML3* (refs. ^11,12^). To test whether the demethylases were responsible for the H3K4me3-mediated loss of CG methylation, we transformed SDG2cd–ZF into the *ros1-3 dml2-1 dml3-1* (*rdd)* triple mutant^35^. We found that none of 40 SDG2cd–ZF T1 lines in this background showed reduced DNA methylation at the *FWA* locus as measured by an McrBC-qPCR assay (Fig. 4a), indicating that the presence of demethylases is required for methylation loss. To rule out the possibility that this result is attributable to SDG2cd–ZF transgene silencing caused by hypermethylation in the *rdd* mutant background, we performed western blot analysis to examine the protein expression levels of SDG2cd–ZF in both the *rdd* mutant and Col-0 backgrounds. While the overall expression levels of SDG2cd–ZF in the *rdd* mutant background were slightly lower compared with the Col-0 background (Extended Data Fig. 4a), several T1 lines in the *rdd* mutant showed higher or comparable expression levels of SDG2cd–ZF compared with the Col-0 background (Extended Data Fig. 4b). Even in these highly expressing SDG2cd–ZF lines, the removal of DNA methylation was completely blocked in the *rdd* background (Fig. 4a,b and Extended Data Fig. 4b). To examine whether the removal of DNA methylation was also blocked by the *rdd* mutant across ZF off-target sites, WGBS experiments were conducted in the *rdd* mutant compared with SDG2cd–ZF in the *rdd* background. The *rdd* mutant effectively blocked DNA methylation loss in all contexts (CG, CHG and CHH) within gene bodies, transposable elements and intergenic regions at ZF off-target sites (Extended Data Figs. 3a,b and 4c).

Previous studies have shown that the histone acetyltransferase IDM1 (Increased DNA Methylation 1) plays a role in DNA demethylation at a subset of loci targeted by ROS1^36,37^. To investigate whether IDM1 is necessary for SDG2cd–ZF-mediated DNA demethylation at the *FWA* locus, we introduced SDG2cd–ZF into the *idm1* mutant background. In contrast to the *rdd* mutant, the *idm1* mutant failed to block the removal of DNA methylation at *FWA* locus (Extended Data Fig. 4d), suggesting that IDM1 is not required for the DNA demethylation at *FWA* locus in SDG2cd–ZF transgenic lines. Together, these results indicate targeting of H3K4me3 at *FWA* and other genomic sites leads to DNA demethylation via its active removal by ROS1/DML2/DML3 demethylase enzymes.

We next investigated whether SDG2cd–ZF was able to cause activation of *FWA* gene expression in the *rdd* mutant background, despite the lack of DNA methylation removal. We found that SDG2cd–ZF was still capable of inducing some *FWA* activation in *rdd*, albeit at much lower levels compared with the wild-type background (Fig. 4c). Similarly, at the ZF off-target sites, SDG2cd–ZF was still able to induce intergenic transcription in a small number of gene body methylated protein coding genes in the absence of DNA demethylation, as well as induce expression of some methylated transposable elements (Extended Data Fig. 3a,b), although often to a lesser extent than in the Col-0 background. Consistent with these results, ChIP-seq experiments continued to show strong enrichment of H3K4me3 at *FWA* and ZF off-target sites in the *rdd* genetic background (Fig. 4d,e). These results suggest that H3K4me3 deposition at *FWA* and other sites can stimulate transcription via two different mechanisms, one that is dependent on DNA methylation removal and one that is independent. In addition, these results show that, despite the presence of high levels of H3K4me3 at DNA methylated sites in the *rdd* mutant, maintenance of CG methylation appears to operate normally (Extended Data Fig. 3a,b). This further underscores that H3K4me3 antagonizes CG DNA methylation by the active removal of methylation, rather than by affecting DNA methylation maintenance mechanisms.

**Fig. 5:**
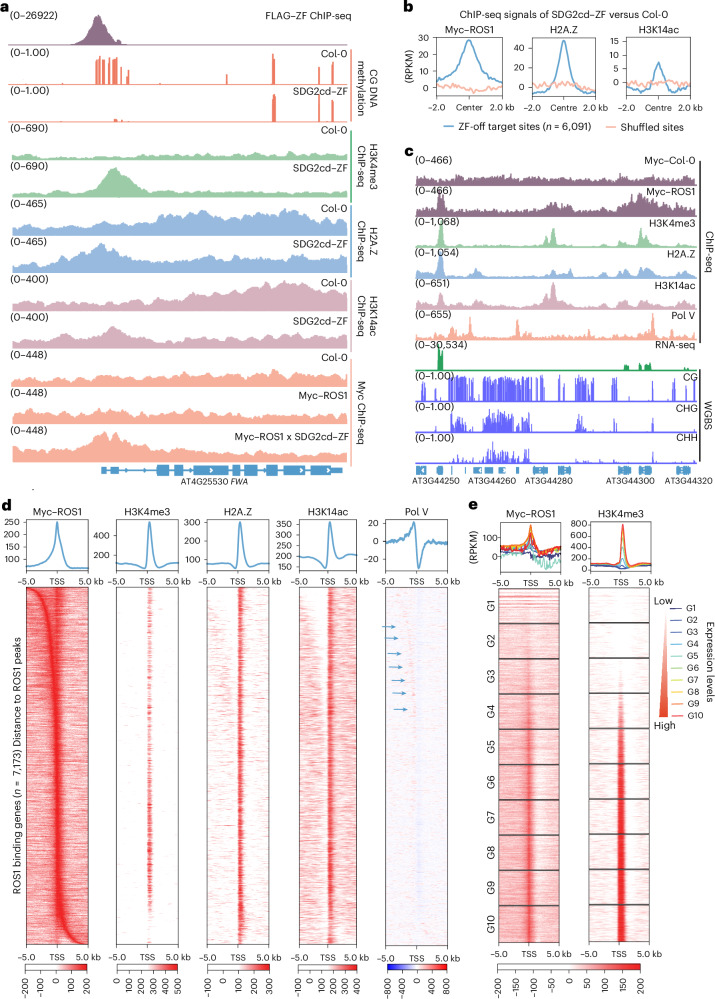
ROS1 accumulated at target genes with higher level of H3K4me3. **a**, Genome browser view of CG DNA methylation, and H3K4me3, H2A.Z and H3K14ac ChIP-seq signals in Col-0 and SDG2cd–ZF transgenic lines at *FWA*, as well as Myc ChIP-seq signals at *FWA* in Col-0 and transgenic lines of Myc–ROS1 or Myc–ROS1xSDG2cd–ZF. **b**, Metaplots indicate the normalized Myc–ROS1, H2A.Z and H3K14ac ChIP-seq signals in SDG2cd–ZF versus Col-0 over ZF off-target and shuffled sites (*n* = 6,091), respectively. **c**, Genome browser view showing ChIP-seq signals of Myc–Col-0, Myc–ROS1, H3K4me3, H3K14ac, H2A.Z, Pol V, RNAseq and WGBS signals of Col-0 over representative ROS1 binding sites. **d**, Metaplots and heat maps depicting the normalized ChIP-seq signals of Myc–ROS1, H3K4me3, H2A.Z, H3K14ac and Pol V over ROS1 bound genes (*n* = 7,173). The arrowheads highlight the enriched ChIP-seq signal of Pol V. **e**, Metaplots and heat maps presenting the normalized ChIP-seq signals of Myc–ROS1 and H3K4me3 for 10 groups of genes sorted by gene expression levels. The numbers in parentheses indicate the data range of the ChIP-seq signals (RPKM) in **a** and **c**.

**Extended Data Fig. 5:**
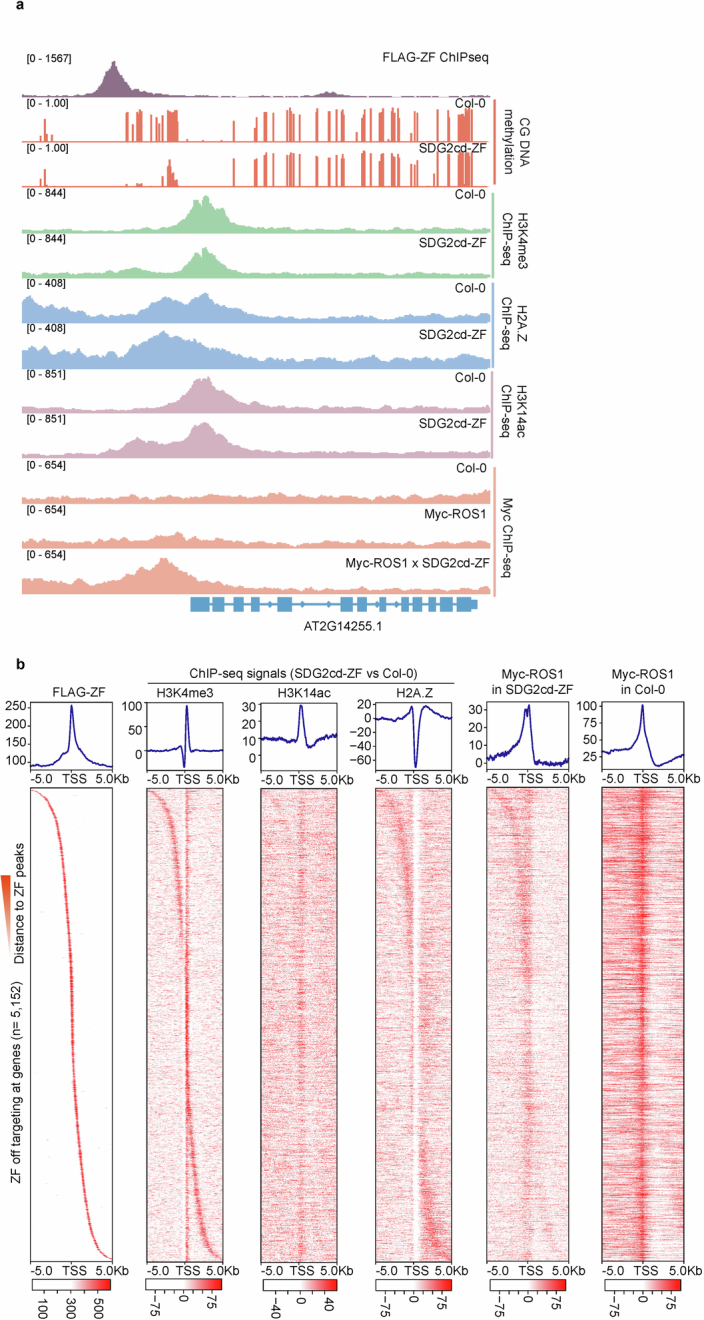
H3K4me3 recruits ROS1 for the removal of DNA methylation. **a**, Genome browser view showing CG DNA methylation, and ChIP-seq signals of H3K4me3, H2A.Z, and H3K14ac in the Col-0 and SDG2cd-ZF transgenic lines, as well as Myc ChIP-seq in the Col-0, Myc-ROS1, and Myc-ROS1xSDG2cd-ZF at a representative ZF off target site. The FLAG-ZF ChIP-seq signal indicates the ZF binding site, and the numbers in parentheses representing the data range of the ChIP-seq signals (RPKM). **b**, Metaplot and heatmaps depicting the ChIP-seq signals of FLAG-ZF, H3K4me3, H3K14ac, H2A.Z, as well as Myc-ROS1 in SDG2cd-ZF and Col-0 background, respectively.

To further verify the involvement of DNA demethylases in the DNA methylation losses induced by SDG2cd–ZF, we transformed Myc–ROS1 into an SDG2cd–ZF transgenic line and performed Myc ChIP-seq. We observed a strong enrichment of ROS1 signal at *FWA* as well as at the ZF off-target sites (Fig. 5a,b and Extended Data Fig. 5a,b), suggesting that H3K4me3 deposition recruits ROS1 to initiate DNA demethylation. It is worth noting that the ROS1 peaks were broader than the H3K4me3 peaks (Fig. 5a and Extended Data Fig. 5a,b), which may explain why the regions of reduced DNA methylation were often wider than the corresponding H3K4me3 enriched regions (Extended Data Fig. 3a,b). Consistent with previous work showing an interaction between ROS1 and the histone variant H2A.Z^36^, as well as the known colocalization of H3K4me3 and H2A.Z at gene promoters^38^, we also found a strong enrichment of H2A.Z over *FWA* and the ZF off-target sites in the SDG2cd–ZF transgenic lines (Fig. 5a,b and Extended Data Fig. 5a,b). Finally, because histone acetylation is normally co-localized with H3K4me3^24,39^, and H3K14 acetylation (H3K14ac) has been shown to be involved in recruitment of the SWR1 complex to deposit H2A.Z^36,37^, we performed H3K14ac ChIP-seq. We found a prominent enrichment of H3K14ac at *FWA* and ZF off-target sites in the SDG2cd–ZF plants (Fig. 5a,b and Extended Data Fig. 5a,b). These results show that SDG2cd–ZF-mediated H3K4me3 deposition promotes the targeting of H3K14ac, H2A.Z and ROS1, together with demethylation of DNA, suggesting that H3K4me3 recruits a suite of activities that have been linked to active DNA demethylation.

The association of SDG2cd-mediated H3K4me3 with the recruitment of ROS1 prompted us to test whether ROS1 is normally associated with peaks of H3K4me3 in the promoters of genes in wild-type plants. We therefore performed Myc–ROS1 ChIP-seq in wild-type plants. We identified ROS1 peaks at loci previously reported to be enriched with ROS1 by ChIP-qPCR (quantitative PCR) (Supplementary Fig. 2)^37^. It is worth noting that we indeed observed strong enrichment of ROS1 at H3K4me3 peaks near the TSSs of genes (Fig. 5c,d). ROS1 was also partially overlapped with other gene-adjacent sites corresponding to sites of DNA methylation and the RdDM factor Pol V (Fig. 5c,d), suggesting that ROS1 is also recruited to these sites where it acts to antagonize DNA methylation as previously reported^11,40^. As expected from previous work^36,37^, we also observed enrichment of H2A.Z and H3K14ac near H3K4me3 peaks at the 5′ end of genes and transposable elements, but not the intergenic regions (Fig. 5c,d and Supplementary Fig. 3). It is worth noting that ROS1 ChIP-seq signals at TSSs were higher at genes with higher expression levels and higher H3K4me3 levels (Fig. 5c,e). These results suggest that ROS1 recruitment to H3K4me3 sites in promoters may normally serve to protect genes from aberrant hypermethylation and that SDG2cd–ZF-mediated recruitment of DNA demethylases may use this natural pathway. These results are also consistent with the virtually perfect non-overlap of H3K4me3 and DNA methylation throughout the genome in wild-type plants^26,41^, as well as the anti-correlation between H2A.Z and DNA methylation^42^.

**Extended Data Fig. 6:**
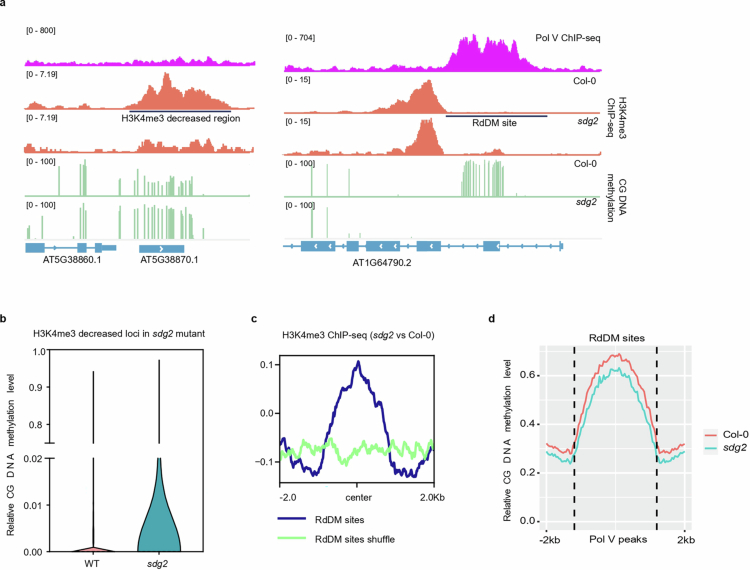
Changes in H3K4me3 in the *sdg2* mutant correlate with changes in CG DNA methylation. **a**, Genome browser views showing Pol V ChIP-seq signal in Col-0, along with H3K4me3 ChIP-seq signals and CG DNA methylation levels in Col-0 and the *sdg2* mutant at two representative loci, respectively. The numbers in the parentheses indicate the data range of the ChIP-seq signals (RPKM). An area is marked in each view where changes in H3K4me3 correspond to changes in CG DNA methylation. **b**, Boxplot showing the average CG DNA methylation level of Col-0 and *sdg2* mutant at regions with reduced H3K4me3 in the *sdg2* mutant. **c**, Metaplot depicting the normalized H3K4me3 ChIP-seq signals in the *sdg2* mutant versus Col-0 at RdDM sites (Pol V ChIP-seq peaks) and shuffled sites. **d**, Metaplot presenting the average CG DNA methylation level at RdDM sites in Col-0 and the *sdg2* mutant, respectively.

To further investigate the effect of H3K4me3 on DNA methylation at endogenous genomic loci, we reanalysed previously reported WGBS and H3K4me3 ChIP-seq data from the *sdg2* mutant^43,44^. We found that CG DNA methylation was mildly increased at regions showing reduced H3K4me3 in the *sdg2* mutant compared with Col-0 (Extended Data Fig. 6a left panel and 6b). It is worth noting that H3K4me3 was mildly redistributed to RdDM regions in the *sdg2* mutant, and this was associated with substantial reduction of DNA methylation at these sites (Extended Data Fig. 6a right panel, and 6c,d). These results are consistent with the gain-of-function findings of SDG2cd–ZF, further confirming the antagonistic relationship between H3K4me3 and DNA methylation.

### Removal of H3K4me3 facilitates targeted DNA methylation

**Fig. 6:**
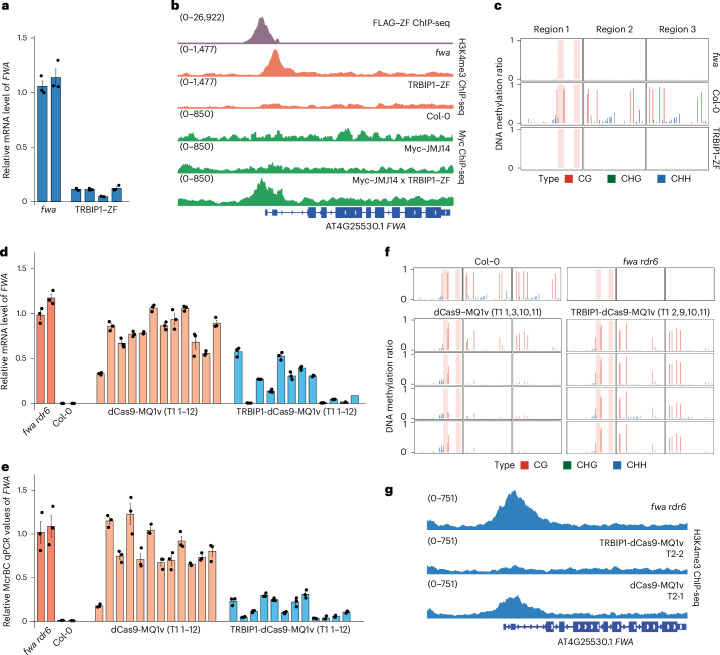
Co-targeting of TRBIP1 and MQ1v resulted in synergistic DNA methylation establishment. **a**, qRT–PCR assay indicating the relative mRNA level of *FWA* in *fwa* and four T2 transgenic lines of TRBIP1–ZF (*n* = 4 biological replicates). **b**, Genome browser view showing H3K4me3 ChIP-seq signals over the *FWA* region in the *fwa* and TRBIP1–ZF transgenic lines, as well as Myc ChIP-seq signals at *FWA* in the Col-0, and transgenic lines of Myc–JMJ14 and Myc–JMJ14xTRBIP1–ZF. FLAG–ZF ChIP-seq signal indicates the ZF binding site. **c**, BS-PCR-seq measuring the CG, CHG and CHH DNA methylation levels of *FWA* promoter regions in *fwa*, Col-0 and transgenic lines of TRBIP1–ZF. **d**, qRT–PCR assay indicating the relative mRNA levels of *FWA* in *fwa rdr6*, Col-0 and T1 transgenic lines of dCas9-MQ1v, TRBIP1-dCas9-MQ1v (*n* = 12 biological replicates). **e**, Relative McrBC-qPCR values at *FWA* in *fwa rdr6*, Col-0 and T1 transgenic lines of dCas9-MQ1v, TRBIP1-dCas9-MQ1v. A lower value indicates a relatively higher level of DNA methylation (*n* = 12 biological replicates). **f**, BS-PCR-seq showing CG, CHG and CHH DNA methylation levels at *FWA* promoter regions in Col-0, *fwa rdr6* and four T1 transgenic lines of dCas9-MQ1v, TRBIP1-dCas9-MQ1v. Pink vertical boxes indicate ZF binding sites. **g**, Genome browser view showing H3K4me3 ChIP-seq signals at the *FWA* region in *fwa rdr6*, dCas9-MQ1v and TRBIP1-dCas9-MQ1v transgenic lines. The numbers in parentheses indicate the data range of the ChIP-seq signals (RPKM) in **b** and **g**. Data are presented as mean values ± s.e.m. in **a**, **d** and **e**.

**Extended Data Fig. 7:**
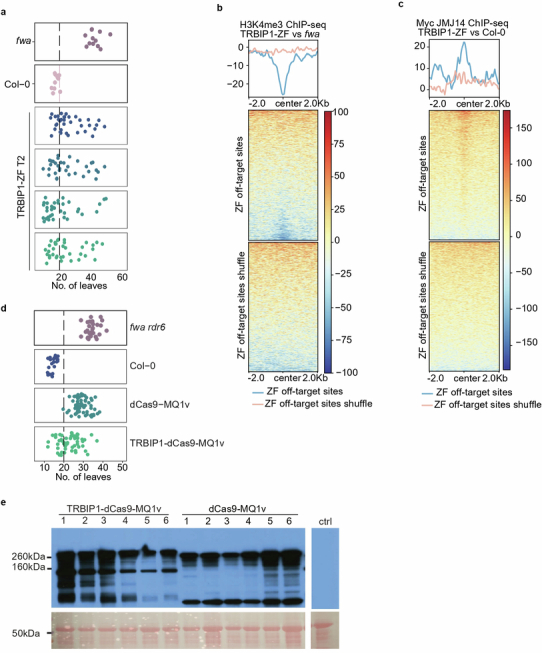
TRBIP1 facilitated the targeted DNA methylation by dCas9-MQ1v. **a**, Dot plots representing leaf numbers of *fwa*, Col-0, and four T2 transgenic lines of TRBIP1-ZF. **b**, Metaplot and heatmaps depicting the normalized H3K4me3 ChIP-seq signal over ZF off-target sites and shuffled sites (n = 6,091) in the TRBIP1-ZF transgenic lines versus *fwa*. **c**, Metaplot and heatmaps depicting the normalized Myc ChIP-seq signal in the Myc-JMJ14xTRBIP1-ZF versus Myc-JMJ14 transgenic lines over ZF off-target sites and shuffled sites (n = 6,091). **d**, Dot plots representing leaf numbers of *fwa rdr6*, Col-0, and T1 transgenic lines of dCas9-MQ1v and TRBIP1-dCas9-MQ1v. **e**, Western blotting showing protein expression levels in an *fwa rdr6* control, dCas9-MQ1v, and TRBIP1-dCas9-MQ1v transgenic lines. Source data

Because of the antagonism between H3K4me3 and DNA methylation, we hypothesized that the targeting of H3K4me3 demethylation might facilitate more efficient installation of DNA methylation at gene promoter sequences. We therefore searched for proteins that could potently target H3K4me3 demethylation. We recently reported that ZF fused to the H3K4me3 demethylase JUMONJI14 (JMJ14) caused some loss of H3K4me3 and partial silencing of *FWA* and other ZF bound loci^31^. We also found that ZF fusion with TELOMERE REPEAT BINDING FACTOR1 (TRB1), TRB2 and TRB3 caused partial silencing of *FWA* and other ZF bound loci at least in part through recruitment of JMJ14 and H3K4me3 demethylation^45^. Because these ZF fusions showed only moderate efficiency in gene silencing^31,45^, we looked for other factors capable of inducing gene silencing using a list of proteins found to co-immunoprecipitate with TRBs by immunoprecipitation mass spectrometry (IP-MS)^45^. We identified a small coiled coil domain protein At4g35510, which we named TRB INTERACTING PROTEIN1 (TRBIP1), that showed very potent gene silencing of *FWA* and an early-flowering phenotype when fused with ZF and introduced into the unmethylated *fwa* epiallele background (Fig. 6a and Extended Data Fig. 7a). To investigate the silencing mechanism of TRBIP1, we generated FLAG–TRBIP1 transgenic plants and performed IP-MS. TRBIP1 showed a similar set of interacting proteins to TRBs (Supplementary Table 2), including JMJ14, suggesting that TRBIP1 functions similarly in silencing *FWA* expression by removal of H3K4me3. H3K4me3 ChIP-seq confirmed that TRBIP1–ZF caused very efficient removal of H3K4me3 (Fig. 6b and Extended Data Fig. 7b), to a greater extent than that observed previously for JMJ14–ZF or TRB–ZFs^31,45^, which may be due to the small size of TRBIP1 or its ability to recruit JMJ14 in a transient or repeated manner. BS-PCR-seq analysis showed that, like JMJ14–ZF and TRB–ZFs^31,45^, TRBIP1–ZF did not establish DNA methylation at the *FWA* promoter region (Fig. 6c). In addition, Myc ChIP-seq in Myc–JMJ14×TRBIP1–ZF lines showed that JMJ14 was strongly recruited to *FWA* and ZF off-target sites (Fig. 6b and Extended Data Fig. 7c). Together, these results show that TRBIP1–ZF targets efficient gene silencing, JMJ14 recruitment and H3K4me3 removal.

To test whether forced H3K4me3 removal could increase the efficiency of targeted DNA methylation, we combined the targeting of the CG specific SssI/MQ1 bacterial methyltransferase with TRBIP1. We previously showed that when an amino acid variant of MQ1 with improved specificity (MQ1v) was fused with dCas9 (ref. ^46^), it mediated rather inefficient targeting of DNA methylation and silencing at *FWA*^23^. To test whether the addition of TRBIP1 could improve this efficiency, we created TRBIP1-dCas9-MQ1v and compared this fusion with the original dCas9-MQ1v. Constructs were transformed into the unmethylated *fwa* epiallele background that also contained the *rdr6* mutation to reduce transgene silencing^31^. We found that the addition of TRBIP1 triggered a strong and consistent increase in DNA methylation, reduced H3K4me3, robustly silenced *FWA* and triggered an early-flowering phenotype (Fig. 6d–g and Extended Data Fig. 7d). To ensure that this effect was not due to differences in protein expression levels, we compared the expression of TRBIP1-dCas9-MQ1v with dCas9-MQ1v and found that they were very similar (Extended Data Fig. 7e). These data show that the removal of H3K4me3 can enhance the deposition of DNA methylation.

## Discussion

This work shows that targeting H3K4me3 to specific regions of the *Arabidopsis* genome results in DNA demethylation at these loci that is dependent on the ROS1 class of DNA demethylases. This demethylation was associated with the recruitment of ROS1, together with the histone variant H2A.Z and histone acetylation. At most locations in the genome, H3K4me3 targeting caused a loss of DNA methylation that was not associated with an increase in transcription, showing that the loss of DNA methylation was not an indirect effect of stimulation of transcription. Furthermore, we found that the targeting of H3K4me3 caused robust recruitment of the ROS1 demethylase enzyme. ROS1 was also found to be naturally present at H3K4me3 sites in the promoters of protein coding genes, which may help to explain the lack of DNA methylation in the majority of gene promoters. The nearly ubiquitous presence of H3K4me3 at active gene promoters likely serves in part to prevent protein coding genes from gaining methylation and being silenced over time^26^. H3K4me3 is thus a powerful anti-DNA methylation mark that is involved in shaping methylation patterns throughout the genome.

Although the ROS1 class of demethylases are specific to plants, the antagonism of H3K4me3 and DNA methylation in *Arabidopsis* described here is reminiscent of mammalian methylation systems, in which the de novo DNA methylation factor Dnmt3L is recruited to unmethylated H3K4 sites and repelled by H3K4me3^47–49^. Therefore, H3K4me3 serves as an anti-DNA methylation mark in a variety of eukaryotic systems, even though the mechanisms for this can be very different.

An understanding of the antagonistic relationship between H3K4me3 and DNA methylation should facilitate the development of more sophisticated tools for the manipulation of DNA methylation patterns in plant genomes. Indeed, we demonstrated that combining an H3K4me3 removal factor with a DNA methyltransferase produced more efficient DNA methylation establishment in *Arabidopsis*. Targeted genome methylation can be used to modulate gene expression and create novel epialleles for plant research and agricultural biotechnology^23,50,51^. The concepts outlined in this work may prove useful in designing more efficient methylation targeting systems in crop plants for the development of epialleles for important plant traits.

## Methods

### Plant materials and growth conditions

All plants used in this study were Columbia-0 (Col-0) ecotype and were grown on soil under long-day conditions (16 h of light and 8 h of dark) at approximately 25 °C. The *rdr6-15*, *fwa rdr6-15*^31^, *idm1* (SALK_062999C) and *rdd* (*ros1-3, dml2-1, dml3-1*)^35^ mutant lines were described previously, and the transgenic lines were generated using floral dipping by *Agrobacterium*. For all the transgenic lines, we obtained more than 36 individual T1 transgenic lines.

### Plasmid construction

For the construction of SDG2cd–ZF, the coding sequence (CDS) of SDG2cd (from the 1,571st to 2,335th amino acids) was cloned into pENTR/D-TOPO vectors (catalogue number K240020, Thermo Fisher), then to the destination vectors MDC123 by using the LR Clonase II Enzyme mix (catalogue number 11791020, Thermo Fisher), which contains the UBQ10 promoter, as well as ZF108 and 3xFLAG fused in the C terminal of the SDG2cd, with hygromycin and basta resistance in plants, respectively. For the construction of SDG2cd(H1866K)–ZF, ENTR-SDG2cd was used as template to generated the H1866K site mutation version of ENTR-SDG2cd(H1866K), and it was then cloned into the destination vector MDC123 containing ZF108 and 3xFLAG. For the construction of SunTag–SDG2cd, Previous SunTag vector was digested by BsiWI (catalogue number ER0851, Thermo Fisher)^23^ and used for infusion reaction with SDG2cd (catalogue number 639650, Takara). For the construction of TRBIP1–ZF, The CDS sequence of TRBIP1 (AT4G35510) was cloned into pENTR/D-TOPO vectors (catalogue number K240020, Thermo Fisher), then to the destination vectors MDC123 by the LR reaction (catalogue number 11791020, Thermo Fisher). For the construction of TRBIP1-dCas9-MQ1v, the CDS of TRBIP1 and MQ1v were amplified^23^, respectively, and ligated with SV40 linker by overlapping PCR.

### Flowering time measurement

Flowering time was determined by the total number of leaves, which included both rosette and caulinar leaves on each plant. The position of each dot in the dot plots represent the leaf counts of different plants.

### Statistics and reproducibility

The western blot experiments in the Extended Data Figs. 1a, 2b, 4a,b and 7e were independently repeated twice with similar results.

### McrBC-qPCR

Relative DNA methylation level at *FWA* locus can be quantified by McrBC-qPCR. McrBC (catalogue number M0272L, NEB) is a restriction endonuclease that recognizes and cleaves DNA sequences with 5-methylcytosine. Equal amounts of DNA were incubated with either McrBC or water (as a control) at 37 °C for 4 h, followed by inactivation at 65 °C for 20 min. The DNA was then used as a template for qPCR to amplify the *FWA* locus using a specific pair of primers (forward TTGGGTTTAGTGTTTACTTG and reverse GAATGTTGAATGGGATAAGGTA). A lower relative McrBC-PCR value indicates a higher level of DNA methylation.

### BS-PCR-seq

Four- to five-week-old *Arabidopsis* leaf samples were collected and prepared for cetyl trimethyl ammonium bromide-based DNA extraction. A total of 2 µg DNA of each sample was used to perform bisulfite treatment by following the manual of the EpiTech Bisulfite kit (catalogue number 59104, QIAGEN). Next, the PCR reactions were performed using the converted DNA as a template to amplify three regions located at *FWA* promoter: region 1 (chr4: 13038143-13038272), region 2 (chr4: 13038356-13038499) and region 3 (chr4: 13038568-13038695). A special polymerase Pfu Turbo Cx (catalogue number 600410, Agilent) was used, and the primers were listed in Supplementary Table 3 (ref. ^45^). The PCR products from regions 1–3 of each sample were pooled and purified using AMPure beads (catalogue number A63881, Beckman Coulter), which were then subjected to library construction using Kapa DNA Hyper Kit (catalogue number KK8502, Roche) and TruSeq DNA UD indexes for Illumina (Illumina). The BS-PCR-seq libraries were sequenced on Illumina iSeq 100.

### ChIP-seq

The ChIP-seq was performed as described previously^31^. About 2–3 g of leaf tissue were collected and ground with liquid nitrogen. The resulting powder was subsequently resuspended in 25 ml of nuclear isolation buffer (50 mM Hepes, 1 M sucrose, 5 mM KCl, 5 mM MgCl_2_, 0.6% Triton X-100, 0.4 mM PMSF, 5 mM benzamidine, 1 Protease Inhibitor Cocktail (catalogue number 11873580001, Sigma)) containing 1% formaldehyde and then agitated to facilitate crosslinking at room temperature for 10 min. Freshly made 1.7 ml 2 M glycine was added to end the crosslinking procedure. The resuspended samples were filtered through a single-layer Miracloth (catalogue number 475855-1R, EMD millipore) and centrifuged at 2,880 × *g* at 4 °C for 20 min. The pellets were resuspended with extraction buffer 2 (0.25 M sucrose, 10 mM Tris–HCl pH = 8.0, 10 mM MgCl_2_, 1% Triton X-100, 5 mM β-mercaptoethanol, 0.1 mM PMSF, 5 mM benzamidine, 1 Protease Inhibitor Cocktail) in 2 ml Eppendorf tube, which was centrifuged again at 12,000 × *g* at 4 °C for 10 min. The pellets were further resuspended with extraction buffer 3 (1.7 M sucrose, 10 mM Tris–HCl pH = 8.0, 2 mM MgCl_2_, 0.15% Triton X-100, 5 mM β-mercaptoethanol, 0.1 mM PMSF, 5 mM benzamidine, 1× Protease Inhibitor Cocktail) and centrifuged at 12,000 × *g* at 4 °C for 1 h. Then the pellets were resuspended with 400 μl lysis buffer (50 mM Tris–HCl pH = 8.0, 10 mM EDTA, 1% SDS, 0.1 mM PMSF, 5 mM benzamidine, 1× cOmplete, Mini, EDTA-free Protease Inhibitor Cocktail (catalogue number 11836170001, Sigma)), diluted with 1.7 ml ChIP dilution buffer (1.1% Triton X-100, 1.2 mM EDTA, 16.7 mM Tris–HCl pH = 8.0, 167 mM NaCl, 0.1 mM PMSF, 5 mM benzamidine, 1 Protease Inhibitor Cocktail) and sheared by Bioruptor Plus (catalogue number B01020001, Diagenode) for 23 cycles (each cycle consisting of 30 s on and 30 s off). The lysate was centrifuged with max speed at 4 °C for 10 min, and the supernatant was carefully transferred to new tubes. This procedure was repeated twice, and the supernatant was incubated with the respective antibody at 4 °C overnight. About 25 μl magnetic Protein A and Protein G Dynabeads (catalogue number 10002D and 10004D, Invitrogen) was added, and the mixture was incubated at 4 °C for 2 h. Next, the beads were washed at 4 °C for 5 min with low-salt solution (150 mM NaCl, 0.2% SDS, 0.5% Triton x-100, 2 mM EDTA, 20 mM Tris–HCl pH = 8.0) (twice), high-salt solution (500 mM NaCl, 0.2% SDS, 0.5% Triton x-100, 2 mM EDTA, 20 mM Tris–HCl pH = 8.0), LiCl solution (250 mM LiCl, 1% Igepal, 1% sodium deoxycholate, 1 mM EDTA, 10 mM Tris–HCl pH = 8.0) and TE solution (10 mM Tris–HCl pH = 8.0 and 1 mM EDTA) at 4 °C for 5 min with each solution. The beads were eluted with 500 μl elution buffer (1% SDS, 10 mM EDTA, 0.1 M NaHCO_3_) at 65 °C for 30 min, and reverse crosslinking was performed at 65 °C overnight. Then 1 μl of 20 mg ml^−1^ Protease K, 10 μl of 0.5 M EDTA pH 8.0 and 20 μl 1 M Tris pH 6.5 were added for the protein deactivation at 45 °C for 4 h, and the DNA was isolated with 550 μl phenol/chloroform/isoamyl alcohol (25:24:1, catalogue number 15593049, Invitrogen) and 500 μl chloroform using phase lock gel (catalogue number 2302820, VWR) and precipitated with 50 μl of 3 M sodium acetate, 2 μl GlycoBlue (catalogue number AM9516, Invitrogen) and 1 ml of 100% ethanol at −20 °C overnight. The DNA was precipitated by max speed centrifuge at 4 °C for 30 min, and the DNA pellet was washed with 700 μl 70% ethanol and eluted with 10 μl nuclease-free water, which was subjected to library construction using the Ovation Ultra Low System V2 kit (catalogue number 0344NB-A01, NuGEN). The libraries were sequenced on an Illumina NovaSeq 6000 sequencer.

### WGBS

The plant DNA was extracted using DNeasy Plant Kit (catalogue number 69106, Qiagen). A total of 500 ng plant DNA was used for WGBS. The DNA was sheared at 4 °C for 2 min using Covaris. Then fragmented DNA was subjected to end repair and adapter ligation following the manual of the Kapa DNA Hyper Kit (catalogue number KK8502, Roche). The TruSeq DNA UD indexes (catalogue number 20022370, Illumina) were used as adapters. Next, the DNA ligation product was purified with AMPure beads (catalogue number A63881, Beckman Coulter), followed by DNA conversion using EpiTech Bisulfite kit (catalogue number 59104, QIAGEN). The converted DNA and the universal primers from Kapa DNA Hyper Kit were used to construct the libraries and subsequently sequenced on Illumina NovaSeq 6000 sequencer^31^.

### RNA sequencing

Plant RNA was isolated from 4-week-old *Arabidopsis* leaves using the Direct-zol RNA MiniPrep kit (catalogue number R2052, Zymo Research). To construct the RNA-sequencing (RNA-seq) library, 1 µg total RNA per sample was used following the instructions provided in the TruSeq Stranded mRNA kit (catalogue number 20020594, Illumina). The libraries were then sequenced on Illumina NovaSeq 6000 sequencer.

## Quantification and statistical analysis

### ChIP-seq analysis

The ChIP-seq raw reads were filtered and trimmed using trim_galore (v0.6.5), and then mapping to the reference genome (TAIR10) was done with Bowtie2 (v2.1.0)^52^ with default parameters. The duplicated reads were removed using Samtools (v1.9)^53^, and the tracks were generated using deeptools (v3.1.3)^54^. MACS2 (v2.2.1) was used to call the peaks^55^. To calculate the enrichment of H3K4me3, H3K14ac and H2A.Z in ZF transgenic lines versus control lines, the histone ChIP-seq signals were first normalized to their respective inputs using bigwigCompare (deeptools_v3.1.3). Then the normalized histone ChIP-seq signals of the ZF transgenic lines were adjusted by subtracting the signals of the control lines, which were subjected to metaplot and heat map analysis over ZF off-target sites and shuffled sites^31^. A similar method was also applied to analyse the enrichment of Myc ChIP-seq signals in Myc–ROS1×SDG2cd–ZF versus Myc–ROS1 and Myc–JMJ14×TRBIP1–ZF versus Myc–JMJ14.

Upon examining the genome browser, we found that some regions in SDG2cd–ZF did not have clear H3K4me3 peaks, while still being retained by the peak-calling pipeline. It is worth noting that the majority of these regions were located in hypermethylated pericentromeric areas. To ensure accuracy of the analysis, in Fig. 3d and Supplementary Fig. 1, we removed these regions to generate the violin plots and heat maps.

### WGBS and BS-PCR-seq analysis

Analysis of WGBS data was conducted following the pipeline outlined previously^31^. The raw paired-end sequencing reads from each sample were mapped to *Arabidopsis* reference genome TAIR10 using BSMAP (v2.90)^56^, which allowed up to two mismatches and one best hit. To ensure data quality, reads containing more than three consecutive methylated CHH sites were excluded. The methylation level for each cytosine was determined by calculating the ratio of methylated cytosines (C) to the sum of methylated cytosines and unmethylated cytosines: C/(C + T).

To perform BS-PCR-seq analysis, the methylation data within three predefined *FWA* promoter regions were retained to make plots using customized R scripts.

### RNA-seq analysis

The raw reads were mapped to the reference genome of *Arabidopsis* TAIR10 using Bowtie2 (v2.1.0)^52^. RSEM (v1.3.1) was used to calculate the gene expression level using default settings^57^, and Trinity (v2.8.5) was used to call differentially expressed genes (DEGs) with log_2_ FC ≥ 1 and FDR < 0.05 as a cut-off^58^. The track files were generated using Samtools (v1.9) and deeptools (v3.1.3)^53,54^. Region-associated DEG analysis was performed using the online tool available at https://labw.org/rad (ref. ^59^). Briefly, the up- and down-regulated DEGs of TRBIP–ZFs versus *fwa* were used as inputs, and FLAG–ZF ChIP-seq peaks were used as targeting regions to run the program.

### Reporting summary

Further information on research design is available in the Nature Portfolio Reporting Summary linked to this article.

## Supplementary information

Supplementary Information Supplementary Figs. 1–3.

Reporting Summary

Supplementary Table 1 CG DNA methylation and H3K4me3 ChIP-seq data of SDG2cd–ZF and Col-0 at ZF off-target sites, FLAG–TRBIP1 IP-MS and primer sequences.

## Source data

Source Data Extended Data Fig. 1 Unprocessed western blots for Extended Data Fig. 1a.

Source Data Extended Data Fig. 2 Unprocessed western blots for Extended Data Fig. 2b.

Source Data Extended Data Fig. 4 Unprocessed western blots for Extended Data Fig. 4a,b.

Source Data Extended Data Fig. 7 Unprocessed western blots for Extended Data Fig. 7e.

## Data Availability

The high-throughput sequencing data generated in this paper have been deposited in the Gene Expression Omnibus (GEO) database (accession number GSE245961). Source data are provided with this paper.

## References

[1] Jones, P. A. Functions of DNA methylation: islands, start sites, gene bodies and beyond. *Nat. Rev. Genet.* **13**, 484–492 (2012). 22641018 10.1038/nrg3230

[2] Law, J. A. & Jacobsen, S. E. Establishing, maintaining and modifying DNA methylation patterns in plants and animals. *Nat. Rev. Genet.* **11**, 204–220 (2010). 20142834 10.1038/nrg2719 PMC3034103

[3] Soppe, W. J. et al. The late flowering phenotype of fwa mutants is caused by gain-of-function epigenetic alleles of a homeodomain gene. *Mol. Cell* **6**, 791–802 (2000). 11090618 10.1016/s1097-2765(05)00090-0

[4] Zhang, H., Lang, Z. & Zhu, J. K. Dynamics and function of DNA methylation in plants. *Nat. Rev. Mol. Cell Biol.* **19**, 489–506 (2018). 29784956 10.1038/s41580-018-0016-z

[5] Jackson, J. P., Lindroth, A. M., Cao, X. & Jacobsen, S. E. Control of CpNpG DNA methylation by the KRYPTONITE histone H3 methyltransferase. *Nature* **416**, 556–560 (2002). 11898023 10.1038/nature731

[6] Du, J. et al. Dual binding of chromomethylase domains to H3K9me2-containing nucleosomes directs DNA methylation in plants. *Cell* **151**, 167–180 (2012). 23021223 10.1016/j.cell.2012.07.034 PMC3471781

[7] Stroud, H. et al. Non-CG methylation patterns shape the epigenetic landscape in *Arabidopsis*. *Nat. Struct. Mol. Biol.* **21**, 64–72 (2014). 24336224 10.1038/nsmb.2735 PMC4103798

[8] Niederhuth, C. E. & Schmitz, R. J. Putting DNA methylation in context: from genomes to gene expression in plants. *Biochim. Biophys. Acta Gene Regul. Mech.* **1860**, 149–156 (2017). 27590871 10.1016/j.bbagrm.2016.08.009 PMC5203807

[9] Saze, H., Mittelsten Scheid, O. & Paszkowski, J. Maintenance of CpG methylation is essential for epigenetic inheritance during plant gametogenesis. *Nat. Genet.* **34**, 65–69 (2003). 12669067 10.1038/ng1138

[10] Mathieu, O., Reinders, J., Caikovski, M., Smathajitt, C. & Paszkowski, J. Transgenerational stability of the *Arabidopsis* epigenome is coordinated by CG methylation. *Cell* **130**, 851–862 (2007). 17803908 10.1016/j.cell.2007.07.007

[11] Gong, Z. et al. ROS1, a repressor of transcriptional gene silencing in *Arabidopsis*, encodes a DNA glycosylase/lyase. *Cell* **111**, 803–814 (2002). 12526807 10.1016/s0092-8674(02)01133-9

[12] Choi, Y. et al. DEMETER, a DNA glycosylase domain protein, is required for endosperm gene imprinting and seed viability in *Arabidopsis*. *Cell* **110**, 33–42 (2002). 12150995 10.1016/s0092-8674(02)00807-3

[13] Liu, R. & Lang, Z. The mechanism and function of active DNA demethylation in plants. *J. Integr. Plant Biol.* **62**, 148–159 (2020). 31628716 10.1111/jipb.12879

[14] Berr, A. et al. *Arabidopsis* SET DOMAIN GROUP2 is required for H3K4 trimethylation and is crucial for both sporophyte and gametophyte development. *Plant cell* **22**, 3232–3248 (2010). 21037105 10.1105/tpc.110.079962 PMC2990135

[15] Guo, L., Yu, Y., Law, J. A. & Zhang, X. SET DOMAIN GROUP2 is the major histone H3 lysine [corrected] 4 trimethyltransferase in *Arabidopsis*. *Proc. Natl Acad. Sci. USA* **107**, 18557–18562 (2010). 20937886 10.1073/pnas.1010478107 PMC2972934

[16] Lu, F., Cui, X., Zhang, S., Liu, C. & Cao, X. JMJ14 is an H3K4 demethylase regulating flowering time in *Arabidopsis*. *Cell Res.* **20**, 387–390 (2010). 20177424 10.1038/cr.2010.27

[17] Xiao, J., Lee, U. S. & Wagner, D. Tug of war: adding and removing histone lysine methylation in *Arabidopsis*. *Curr. Opin. Plant Biol.* **34**, 41–53 (2016). 27614255 10.1016/j.pbi.2016.08.002

[18] Liu, P. et al. The histone H3K4 demethylase JMJ16 represses leaf senescence in *Arabidopsis*. *Plant Cell* **31**, 430–443 (2019). 30712008 10.1105/tpc.18.00693 PMC6447021

[19] Yang, H. et al. Overexpression of a histone H3K4 demethylase, JMJ15, accelerates flowering time in *Arabidopsis*. *Plant Cell Rep.* **31**, 1297–1308 (2012). 22555401 10.1007/s00299-012-1249-5

[20] Jiang, D., Yang, W., He, Y. & Amasino, R. M. *Arabidopsis* relatives of the human lysine-specific demethylase1 repress the expression of FWA and FLOWERING LOCUS C and thus promote the floral transition. *Plant Cell* **19**, 2975–2987 (2007). 17921315 10.1105/tpc.107.052373 PMC2174716

[21] Mori, S. et al. Cotranscriptional demethylation induces global loss of H3K4me2 from active genes in *Arabidopsis*. *EMBO J.* **42**, e113798 (2023). 37849386 10.15252/embj.2023113798 PMC10690457

[22] Inagaki, S., Takahashi, M., Takashima, K., Oya, S. & Kakutani, T. Chromatin-based mechanisms to coordinate convergent overlapping transcription. *Nat. Plants* **7**, 295–302 (2021). 33649596 10.1038/s41477-021-00868-3

[23] Ghoshal, B., Picard, C. L., Vong, B., Feng, S. & Jacobsen, S. E. CRISPR-based targeting of DNA methylation in Arabidopsis thaliana by a bacterial CG-specific DNA methyltransferase. *Proc. Natl Acad. Sci. USA* **118**, e2125016118 (2021). 34074795 10.1073/pnas.2125016118 PMC8201958

[24] Liu, W. et al. Ectopic targeting of CG DNA methylation in *Arabidopsis* with the bacterial SssI methyltransferase. *Nat. Commun.* **12**, 3130 (2021). 34035251 10.1038/s41467-021-23346-y PMC8149686

[25] Gallego-Bartolome, J. et al. Co-targeting RNA polymerases IV and V promotes efficient de novo DNA methylation in *Arabidopsis*. *Cell* **176**, 1068–1082.e19 (2019). 30739798 10.1016/j.cell.2019.01.029 PMC6386582

[26] Zhang, X., Bernatavichute, Y. V., Cokus, S., Pellegrini, M. & Jacobsen, S. E. Genome-wide analysis of mono-, di- and trimethylation of histone H3 lysine 4 in *Arabidopsis thaliana*. *Genome Biol.* **10**, R62 (2009). 19508735 10.1186/gb-2009-10-6-r62 PMC2718496

[27] Cokus, S. J. et al. Shotgun bisulphite sequencing of the *Arabidopsis* genome reveals DNA methylation patterning. *Nature* **452**, 215–219 (2008). 18278030 10.1038/nature06745 PMC2377394

[28] Zhang, X. et al. Genome-wide high-resolution mapping and functional analysis of DNA methylation in *Arabidopsis*. *Cell* **126**, 1189–1201 (2006). 16949657 10.1016/j.cell.2006.08.003

[29] Johnson, L. M. et al. SRA- and SET-domain-containing proteins link RNA polymerase V occupancy to DNA methylation. *Nature* **507**, 124–128 (2014). 24463519 10.1038/nature12931 PMC3963826

[30] Papikian, A., Liu, W., Gallego-Bartolome, J. & Jacobsen, S. E. Site-specific manipulation of *Arabidopsis* loci using CRISPR-Cas9 SunTag systems. *Nat. Commun.* **10**, 729 (2019). 30760722 10.1038/s41467-019-08736-7 PMC6374409

[31] Wang, M. et al. A gene silencing screen uncovers diverse tools for targeted gene repression in *Arabidopsis*. *Nat. Plants* **9**, 460–472 (2023). 36879017 10.1038/s41477-023-01362-8 PMC10027610

[32] Soares, L. M., Radman-Livaja, M., Lin, S. G., Rando, O. J. & Buratowski, S. Feedback control of Set1 protein levels is important for proper H3K4 methylation patterns. *Cell Rep.* **6**, 961–972 (2014). 24613354 10.1016/j.celrep.2014.02.017 PMC3999964

[33] Schlichter, A. & Cairns, B. R. Histone trimethylation by Set1 is coordinated by the RRM, autoinhibitory, and catalytic domains. *EMBO J.* **24**, 1222–1231 (2005). 15775977 10.1038/sj.emboj.7600607 PMC556409

[34] Tanenbaum, M. E., Gilbert, L. A., Qi, L. S., Weissman, J. S. & Vale, R. D. A protein-tagging system for signal amplification in gene expression and fluorescence imaging. *Cell* **159**, 635–646 (2014). 25307933 10.1016/j.cell.2014.09.039 PMC4252608

[35] Penterman, J. et al. DNA demethylation in the *Arabidopsis* genome. *Proc. Natl Acad. Sci. USA* **104**, 6752–6757 (2007). 17409185 10.1073/pnas.0701861104 PMC1847597

[36] Nie, W. F. et al. Histone acetylation recruits the SWR1 complex to regulate active DNA demethylation in *Arabidopsis*. *Proc. Natl Acad. Sci. USA* **116**, 16641–16650 (2019). 31363048 10.1073/pnas.1906023116 PMC6697875

[37] Qian, W. et al. A histone acetyltransferase regulates active DNA demethylation in *Arabidopsis*. *Science* **336**, 1445–1448 (2012). 22700931 10.1126/science.1219416 PMC3575687

[38] Hu, G. et al. H2A.Z facilitates access of active and repressive complexes to chromatin in embryonic stem cell self-renewal and differentiation. *Cell Stem Cell* **12**, 180–192 (2013). 23260488 10.1016/j.stem.2012.11.003 PMC3570599

[39] Wang, Z. et al. Genome-wide mapping of HATs and HDACs reveals distinct functions in active and inactive genes. *Cell* **138**, 1019–1031 (2009). 19698979 10.1016/j.cell.2009.06.049 PMC2750862

[40] Du, X. et al. Molecular basis of the plant ROS1-mediated active DNA demethylation. *Nat. Plants* **9**, 271–279 (2023). 36624257 10.1038/s41477-022-01322-8

[41] Guo, H. et al. The DNA methylation landscape of human early embryos. *Nature* **511**, 606–610 (2014). 25079557 10.1038/nature13544

[42] Zilberman, D., Coleman-Derr, D., Ballinger, T. & Henikoff, S. Histone H2A.Z and DNA methylation are mutually antagonistic chromatin marks. *Nature* **456**, 125–129 (2008). 18815594 10.1038/nature07324 PMC2877514

[43] Stroud, H., Greenberg, M. V., Feng, S., Bernatavichute, Y. V. & Jacobsen, S. E. Comprehensive analysis of silencing mutants reveals complex regulation of the *Arabidopsis* methylome. *Cell* **152**, 352–364 (2013). 23313553 10.1016/j.cell.2012.10.054 PMC3597350

[44] Chen, L. Q. et al. ATX3, ATX4, and ATX5 encode putative H3K4 methyltransferases and are critical for plant development. *Plant Physiol.* **174**, 1795–1806 (2017). 28550207 10.1104/pp.16.01944 PMC5490889

[45] Wang, M. et al. *Arabidopsis* TRB proteins function in H3K4me3 demethylation by recruiting JMJ14. *Nat. Commun.* **14**, 1736 (2023). 36977663 10.1038/s41467-023-37263-9 PMC10049986

[46] Lei, Y. et al. Targeted DNA methylation in vivo using an engineered dCas9-MQ1 fusion protein. *Nat. Commun.* **8**, 16026 (2017). 28695892 10.1038/ncomms16026 PMC5508226

[47] Douillet, D. et al. Uncoupling histone H3K4 trimethylation from developmental gene expression via an equilibrium of COMPASS, Polycomb and DNA methylation. *Nat. Genet.* **52**, 615–625 (2020). 32393859 10.1038/s41588-020-0618-1 PMC7790509

[48] Cedar, H. & Bergman, Y. Linking DNA methylation and histone modification: patterns and paradigms. *Nat. Rev. Genet.* **10**, 295–304 (2009). 19308066 10.1038/nrg2540

[49] Ooi, S. K. et al. DNMT3L connects unmethylated lysine 4 of histone H3 to de novo methylation of DNA. *Nature* **448**, 714–717 (2007). 17687327 10.1038/nature05987 PMC2650820

[50] Veley, K. M. et al. Improving cassava bacterial blight resistance by editing the epigenome. *Nat. Commun.* **14**, 85 (2023). 36604425 10.1038/s41467-022-35675-7 PMC9816117

[51] Cheng, Y., Zhou, Y. & Wang, M. Targeted gene regulation through epigenome editing in plants. *Curr. Opin. Plant Biol.* **80**, 102552 (2024). 38776571 10.1016/j.pbi.2024.102552

[52] Langmead, B. & Salzberg, S. L. Fast gapped-read alignment with Bowtie 2. *Nat. Methods* **9**, 357–359 (2012). 22388286 10.1038/nmeth.1923 PMC3322381

[53] Li, H. et al. The Sequence Alignment/Map format and SAMtools. *Bioinformatics* **25**, 2078–2079 (2009). 19505943 10.1093/bioinformatics/btp352 PMC2723002

[54] Ramirez, F. et al. deepTools2: a next generation web server for deep-sequencing data analysis. *Nucleic Acids Res.* **44**, W160–W165 (2016). 27079975 10.1093/nar/gkw257 PMC4987876

[55] Zhang, Y. et al. Model-based analysis of ChIP-Seq (MACS). *Genome Biol.* **9**, R137 (2008). 18798982 10.1186/gb-2008-9-9-r137 PMC2592715

[56] Xi, Y. & Li, W. BSMAP: whole genome bisulfite sequence MAPping program. *BMC Bioinformatics* **10**, 232 (2009). 19635165 10.1186/1471-2105-10-232 PMC2724425

[57] Li, B. & Dewey, C. N. RSEM: accurate transcript quantification from RNA-seq data with or without a reference genome. *BMC Bioinformatics* **12**, 323 (2011). 21816040 10.1186/1471-2105-12-323 PMC3163565

[58] Grabherr, M. G. et al. Full-length transcriptome assembly from RNA-Seq data without a reference genome. *Nat. Biotechnol.* **29**, 644–652 (2011). 21572440 10.1038/nbt.1883 PMC3571712

[59] Guo, Y. et al. RAD: a web application to identify region associated differentially expressed genes. *Bioinformatics*10.1093/bioinformatics/btab075 (2021). 33532827 10.1093/bioinformatics/btab075

