# Supplementary material for: Histone H3 lysine 4 methylation recruits DNA demethylases to enforce gene expression in Arabidopsis
Source: Nat Plants. 2025 Feb 11;11(2):206–17. doi: 10.1038/s41477-025-01924-y (PMC11842272; doi:10.1038/s41477-025-01924-y)
Supplement: Supplementary file 1 — Supplementary Figs. 1–3. [file 41477_2025_1924_MOESM1_ESM.pdf]

nature plants

Supplementary information

https://doi.org/10.1038/s41477-025-01924-y

Histone H3 lysine 4 methylation recruits DNA demethylases to enforce gene expression in *Arabidopsis*

In the format provided by the authors and unedited

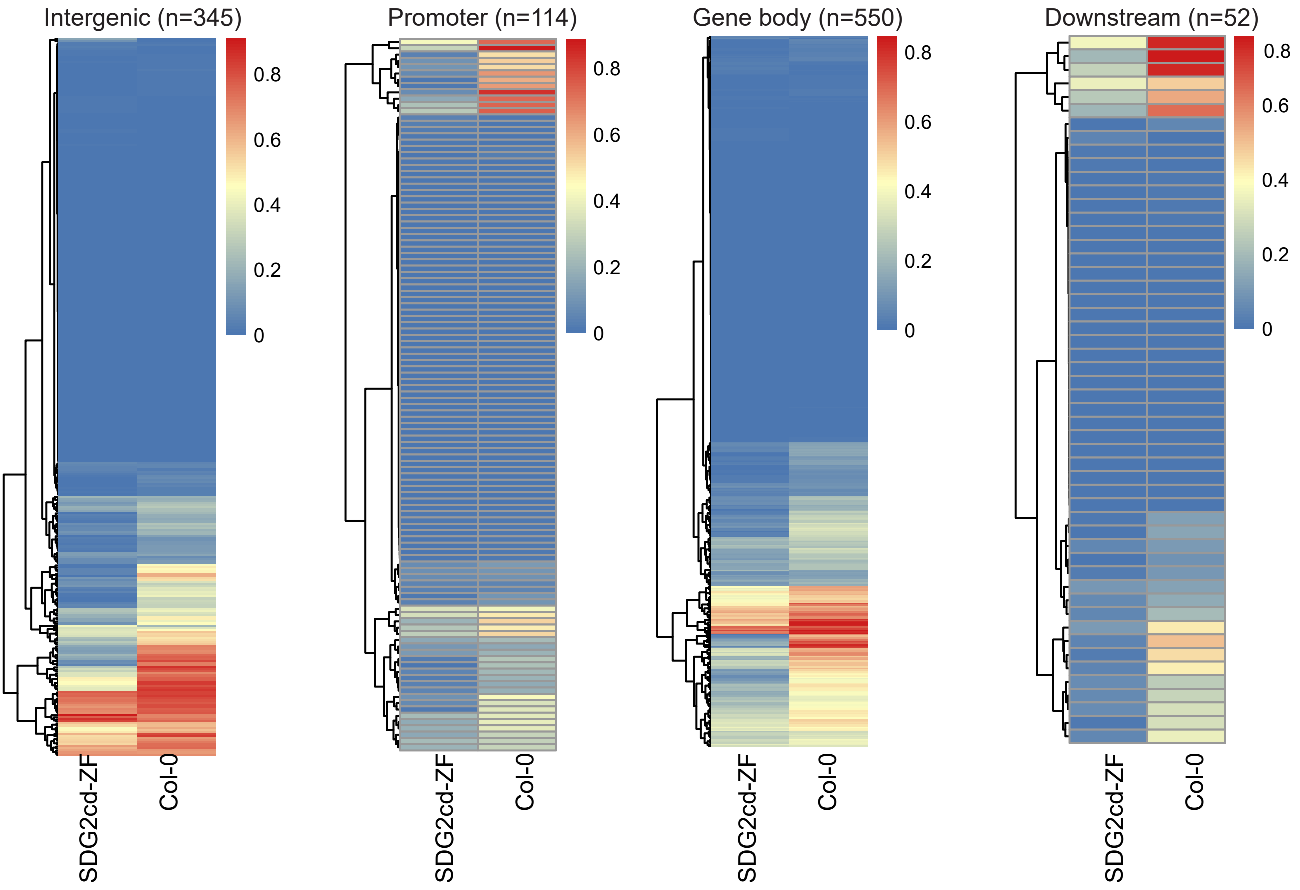

Supplementary Fig. 1 Heatmaps showing the relative CG DNA methylation levels of Col-0 and SDG2cd-ZF across ZF binding genes with strongly increased H3K4me3, spanning the intergenic, promoter, gene body, and downstream regions.

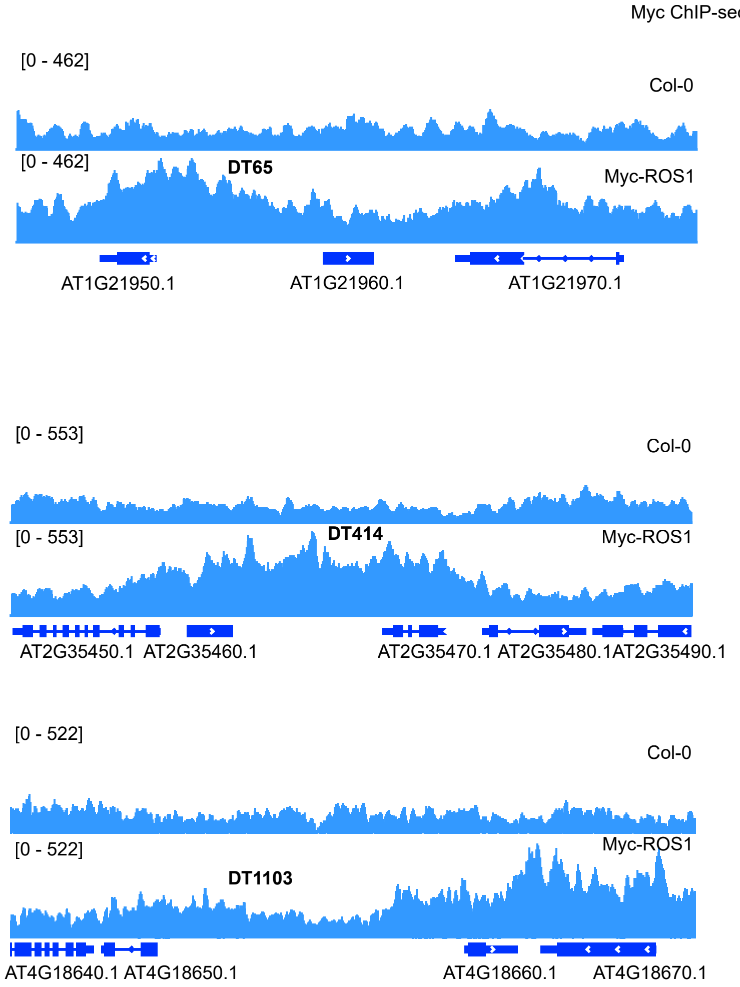

Supplementary Fig. 2 Genome browser views of Myc ChIP-seq signals in Col-0 and Myc-ROS1 at five DT loci previously reported by ChIP-qPCR.

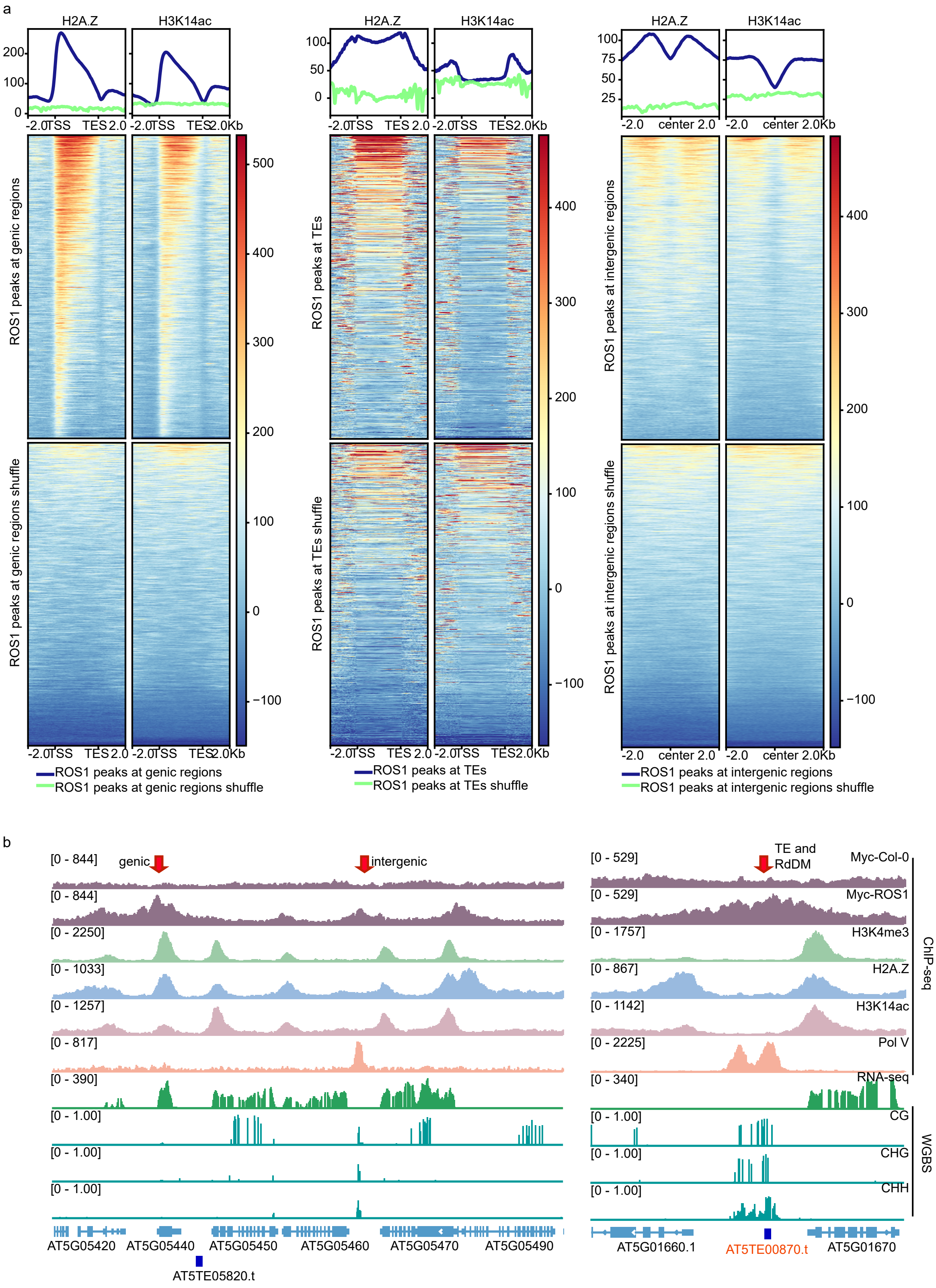

Supplementary Fig. 3 The overlaping of H2A.Z, H3K14ac and ROS1 ChIP-seq signals at different chromatin regions. a, Metaplots and heatmaps displaying the normalized ChIP-seq signals of H2A.Z and H3K14ac at ROS1 targeting genic regions, TEs, intergenic regions and corresponding shuffle sites. **b,** Genome browser view showing ChIP-seq signals of Myc-Col-0, Myc-ROS1, H3K4me3, H3K14ac, H2A.Z, Pol V and RNAseq signal of Col-0 over representative ROS1 binding sites. The red arrowhead indicate the representative examples of ROS1 targeting genic, intergenic and TE regions, respectively.
