# Supplementary material for: Histone H3 lysine 4 methylation recruits DNA demethylases to enforce gene expression in Arabidopsis
Source: Nat Plants. 2025 Feb 11;11(2):206–17. doi: 10.1038/s41477-025-01924-y (PMC11842272; doi:10.1038/s41477-025-01924-y)
Supplement: Supplementary file 2 — Reporting Summary [file 41477_2025_1924_MOESM2_ESM.pdf]

nature portfolio

Corresponding author(s): Steven E. Jacobsen

Last updated by author(s): Jan 11, 2025

Reporting Summary

Nature Portfolio wishes to improve the reproducibility of the work that we publish. This form provides structure for consistency and transparency in reporting. For further information on Nature Portfolio policies, see our Editorial Policies and the Editorial Policy Checklist.

Statistics

For all statistical analyses, confirm that the following items are present in the figure legend, table legend, main text, or Methods section.

n/a	Confirmed	
☐	☒	The exact sample size (*n*) for each experimental group/condition, given as a discrete number and unit of measurement
☐	☒	A statement on whether measurements were taken from distinct samples or whether the same sample was measured repeatedly
☐	☒	The statistical test(s) used AND whether they are one- or two-sided<br>*Only common tests should be described solely by name; describe more complex techniques in the Methods section.*
☒	☐	A description of all covariates tested
☐	☒	A description of any assumptions or corrections, such as tests of normality and adjustment for multiple comparisons
☐	☒	A full description of the statistical parameters including central tendency (e.g. means) or other basic estimates (e.g. regression coefficient) AND variation (e.g. standard deviation) or associated estimates of uncertainty (e.g. confidence intervals)
☐	☒	For null hypothesis testing, the test statistic (e.g. *F*, *t*, *r*) with confidence intervals, effect sizes, degrees of freedom and *P* value noted<br>*Give P values as exact values whenever suitable.*
☒	☐	For Bayesian analysis, information on the choice of priors and Markov chain Monte Carlo settings
☒	☐	For hierarchical and complex designs, identification of the appropriate level for tests and full reporting of outcomes
☒	☐	Estimates of effect sizes (e.g. Cohen's *d*, Pearson's *r*), indicating how they were calculated

Our web collection on statistics for biologists contains articles on many of the points above.

Software and code

Policy information about availability of computer code

Data collection	No software was used for data collection.
Data analysis	RNA-Sequencing analysis<br>The raw reads were mapped to the reference genome of Arabidopsis TAIR10 using Bowtie2 (v2.1.0). RSEM (v1.3.1) was used to calculate the gene expression level using default setting, and Trinity (v2.8.5) was used to call DEGs with log2 FC ≥ 1 and FDR < 0.05 as a cut off. The track files were generated by using Samtools (v1.9) and deeptools (v3.1.3). Region associated DEG analysis (RAD) was performed by using the pipeline as described previously (43). Briefly, the up- and down-regulated DEGs of TRBIP-ZFs versus fwa were used as inputs, and FLAG-ZF ChIP-seq peaks were used as targeting regions to run the pipeline.<br><br>ChIP-seq analysis<br>The ChIP-seq raw reads were filtered and trimmed using trim_galore (v0.6.5), and then mapping to the reference genome (TAIR10) with Bowtie2 (v2.1.0) with default parameters. The duplicated reads were removed by using the Samtools (v1.9), and the tracks were generated by using deeptools (v3.1.3). MACS2 (v2.2.1) was used to call peaks. To calculate the enrichment of H3K4me3, H3K14ac, and H2A.Z in ZF transgenic lines versus control lines, the corresponding histone ChIP-seq signals were first normalized to their respect inputs by using bigwigCompare, the normalized histone ChIP-seq signals of the ZF transgenic lines were further normalized to the control lines by using bigwigCompare, which were subjected to the metaplot and heatmap analysis over ZF off-target sites and shuffled sites. The similar method was also applied to the enrichment of Myc ChIP-seq signals in Myc-ROS1xSDG2cd-ZF versus Myc-ROS1, Myc-JMJ14xTRBIP1-ZF versus Myc-JMJ14. Upon examining the genome browser, we found that some regions in SDG2cd-ZF did not have clear H3K4me3 peaks, while still being retained by the peak-calling pipeline. Notably, the majority of these regions were located in hypermethylated pericentromeric areas. To ensure accuracy of the analysis in Fig. 3d and Supplementary Fig. 1, we removed these regions to generate the violin plots and heatmaps.

WGBS analysis
The analysis of WGBS data was conducted following the pipeline outlined previously. The raw paired-end sequencing reads from each sample were mapped to Arabidopsis reference genome TAIR10 using BSMAP (v2.90), which allowed up to 2 mismatches and 1 best hit. To ensure data quality, the reads with more than 3 consecutive methylated CHH were excluded. The methylation level for each cytosine was determined by calculating the ratio of methylated cytosines (C) to the sum of methylated cytosines and unmethylated cytosines: C/(C+T).
To perform BS-PCR-seq analysis, the methylation data within three predefined FWA promoter regions were retained to make plots by using customized R scripts. R version 4.1.3.

For manuscripts utilizing custom algorithms or software that are central to the research but not yet described in published literature, software must be made available to editors and reviewers. We strongly encourage code deposition in a community repository (e.g. GitHub). See the Nature Portfolio guidelines for submitting code & software for further information.

Data

Policy information about availability of data

All manuscripts must include a data availability statement. This statement should provide the following information, where applicable:

- Accession codes, unique identifiers, or web links for publicly available datasets
- A description of any restrictions on data availability
- For clinical datasets or third party data, please ensure that the statement adheres to our policy

The high-throughput sequencing data generated in this paper have been deposited in the Gene Expression Omnibus (GEO) database (accession no. GSE245961).

Research involving human participants, their data, or biological material

Policy information about studies with human participants or human data. See also policy information about sex, gender (identity/presentation), and sexual orientation and race, ethnicity and racism.

Reporting on sex and gender	N/A
Reporting on race, ethnicity, or other socially relevant groupings	N/A
Population characteristics	N/A
Recruitment	N/A
Ethics oversight	N/A

Note that full information on the approval of the study protocol must also be provided in the manuscript.

Field-specific reporting

Please select the one below that is the best fit for your research. If you are not sure, read the appropriate sections before making your selection.

☒ Life sciences ☐ Behavioural & social sciences ☐ Ecological, evolutionary & environmental sciences

For a reference copy of the document with all sections, see nature.com/documents/nr-reporting-summary-flat.pdf

Life sciences study design

All studies must disclose on these points even when the disclosure is negative.

Sample size	No formal sample size calculation was performed. The sample sizes for RNA-seq, WGBS, ChIP-seq, and BS-PCR experiments were selected based on widely accepted field standards. Specifically, for RNA-seq, we included 3 biological replicates per condition, as recommended by Schurch et al. (2016). For WGBS and ChIP-seq, 2-3 biological replicates were used, following ENCODE consortium guidelines (Landt et al., 2012; Ziller et al., 2013). These sample sizes are considered sufficient to ensure reliable and reproducible results in similar studies.
Data exclusions	No data exclusion in the study.
Replication	Two replicates for ChIP-seq. Two replicates for BS-PCR. Three replicates for RNA-seq samples. Three technical replicates for qRT-PCR. All replicates were performed independently and produced high reproducible results.
Randomization	For all experiments, treatment and control samples were grown side by side, each replicate on separate plate. Allocation of samples were not random, because it is not relevant to the study. Randomization was not applicable in this study because the experiments were designed to investigate specific molecular mechanisms or targeted biological processes, rather than assess randomized groups or conditions. The samples and experimental conditions were predetermined based on the biological context and the study objectives, such as analyzing gene expression changes, DNA methylation and histone modification patterns. These targeted analyses do not involve variability that would necessitate

randomization, as they focus on controlled and reproducible experimental setups.

Blinding

No blinding used because it was largely not relevant to our study. All data were collected based on the genotype of plants, while blinding the samples during the experiments will increases the risk of mislabeling and wrong results.

Reporting for specific materials, systems and methods

We require information from authors about some types of materials, experimental systems and methods used in many studies. Here, indicate whether each material, system or method listed is relevant to your study. If you are not sure if a list item applies to your research, read the appropriate section before selecting a response.

Materials & experimental systems

n/a	Involved in the study
☐	☒ Antibodies
☒	☐ Eukaryotic cell lines
☒	☐ Palaeontology and archaeology
☒	☐ Animals and other organisms
☒	☐ Clinical data
☒	☐ Dual use research of concern
☒	☐ Plants

Methods

n/a	Involved in the study
☐	☒ ChIP-seq
☒	☐ Flow cytometry
☒	☐ MRI-based neuroimaging

Antibodies

Antibodies used

Anti-FLAG Millipore Sigma Cat# F1804; RRID:AB_262044
Anti-FLAG M2-Peroxidase (HRP) Millipore Sigma Sigma-Aldrich Cat# A8592, RRID:AB_439702
Anti-Histone H3 Abcam Cat# ab1791, RRID:AB_302613
Anti-trimethyl-Histone H3 (Lys4)¬ Millipore Sigma Cat# 04-745, RRID:AB_1163444
Anti-Myc-tag (71D10) rabbit mAb Cell Signaling Cat# 2278S, RRID:AB_1658203

Validation

anti-FLAG M2 (Sigma): the antibodies have been validated by the manufacturer, https://www.sigmaaldrich.com/catalog/product/sigma/fl804
Anti-FLAG M2-Peroxidase (HRP)(Sigma): the antibodies have been validated by the manufacturer, https://www.sigmaaldrich.com/US/en/product/sigma/a8592
anti-H3 (Ab1791, Abeam): the antibodies have been validated by the manufacturer, https://www.abcam.com/histone-h3-antibody-nuclear-marker-and-chip-grade-ab1791.html
Anti-H3K4me3 (04-745, Millipore Sigma): the antibodies have been validated by the manufacturer, https://www.emdmillipore.com/US/en/product/Anti-trimethyl-Histone-H3-Lys4-Antibodyclone-MC315-rabbit-monoclona I, MM_NF-04-745
Anti-Myc-tag (71D10) (2278S, Cell Signaling Technology) the antibodies have been validated by the manufacturer, https://www.cellsignal.com/products/primary-antibodies/myc-tag-71d10-rabbit-mab/2278

Plants

Seed stocks

rdr6-15, fwa rdr6-15, idm1-1 and rdd (ros1-3, dml2-1, dml3-1) mutant lines

Novel plant genotypes

SDG2cd-ZF in WT background, SDG2cd-ZF in rdd background, SunTag-SDG2cd in WT background, SunTag-SDG2cd in rdr6 background, TRBIP1-ZF in fwa background, TRBIP2-ZF in fwa background, TRBIP1-dCAS9-MQ1v in fwa rdr6 background.

Authentication

The seeds stocks have been used frequently by multiple previous studies. The novel transgenic lines were compared with a well designed corresponding control lines to avoid secondary effect.

ChIP-seq

Data deposition

☒ Confirm that both raw and final processed data have been deposited in a public database such as GEO.

☒ Confirm that you have deposited or provided access to graph files (e.g. BED files) for the called peaks.

Data access links
May remain private before publication.

The high-throughput sequencing data generated in this paper have been deposited in the Gene Expression Omnibus (GEO) database (accession no. GSE245961 with secure token: wjsbkomqxvidxyh).

Files in database submission

ChIPseq-SET1-H3K4me3-Col0-Rep1_S28_L002.bw
ChIPseq-SET1-H3K4me3-Col0-Rep2_S29_L002.bw
ChIPseq-SET1-H3K4me3-SDG2-ZF-Rep1_S30_L002.bw
ChIPseq-SET1-H3K4me3-SDG2-ZF-Rep2_S31_L002.bw
ChIPseq-SET1-H3K4me3-SDG2-ZF-Rep3_S32_L002.bw
ChIPseq-SET1-input-Col0-Rep1_S43_L002.bw
ChIPseq-SET1-input-Col0-Rep2_S44_L002.bw
ChIPseq-SET1-input-SDG2-ZF-Rep1_S45_L002.bw
ChIPseq-SET1-input-SDG2-ZF-Rep2_S46_L002.bw
ChIPseq-SET1-input-SDG2-ZF-Rep3_S47_L002.bw
ChIPseq-SET10-H3K4me3-TRBIP1-dCAS9-MQ1v-Rep1_S22_L004.bw
ChIPseq-SET10-H3K4me3-TRBIP1-dCAS9-MQ1v-Rep2_S26_L004.bw
ChIPseq-SET10-H3K4me3-dCAS9-MQ1v_S23_L004.bw
ChIPseq-SET10-H3K4me3-fwaxrdr6-Rep1_S21_L004.bw
ChIPseq-SET10-H3K4me3-fwaxrdr6-Rep2_S25_L004.bw
ChIPseq-SET2-H3K4me3-SunTag-SDG2_rdr6-Rep1_S7_L003.bw
ChIPseq-SET2-H3K4me3-SunTag-SDG2_rdr6-Rep2_S8_L003.bw
ChIPseq-SET2-H3K4me3-noguideSDG2_rdr6_S6_L003.bw
ChIPseq-SET2-H3K4me3-rdr6-Rep1_S4_L003.bw
ChIPseq-SET2-H3K4me3-rdr6-Rep2_S5_L003.bw
ChIPseq-SET2-input-SunTag-SDG2_rdr6-Rep1_S25_L003.bw
ChIPseq-SET2-input-SunTag-SDG2_rdr6-Rep2_S26_L003.bw
ChIPseq-SET2-input-noguideSDG2_rdr6_S24_L003.bw
ChIPseq-SET2-input-rdr6-Rep1_S22_L003.bw
ChIPseq-SET2-input-rdr6-Rep2_S23_L003.bw
ChIPseq-SET3-H3K14ac-Col0-Rep1_S11_L003.bw
ChIPseq-SET3-H3K14ac-Col0-Rep2_S12_L003.bw
ChIPseq-SET3-H3K14ac-SDG2-ZF-Rep1_S15_L003.bw
ChIPseq-SET3-H3K14ac-SDG2-ZF-Rep2_S16_L003.bw
ChIPseq-SET3-input-Col0-Rep1_S29_L003.bw
ChIPseq-SET3-input-Col0-Rep2_S30_L003.bw
ChIPseq-SET3-input-SDG2-ZF-Rep1_S31_L003.bw
ChIPseq-SET3-input-SDG2-ZF-Rep2_S32_L003.bw
ChIPseq-SET4-H2AZ-Col0-Rep1_S20_L002.bw
ChIPseq-SET4-H2AZ-SDG2-ZF-Rep1_S21_L002.bw
ChIPseq-SET4-H2AZ-SDG2-ZF-Rep3_S22_L002.bw
ChIPseq-SET4-input-Col0-Rep1_S31_L002.bw
ChIPseq-SET4-input-Col0-Rep2_S32_L002.bw
ChIPseq-SET4-input-ROS1-Rep1_S26_L002.bw
ChIPseq-SET4-input-ROS1xSDG2-ZF-Rep1_S36_L002.bw
ChIPseq-SET4-input-SDG2-ZF-Rep1_S33_L002.bw
ChIPseq-SET4-input-SDG2-ZF-Rep3_S34_L002.bw
ChIPseq-SET4-myc-Col0-Rep1_S19_L002.bw
ChIPseq-SET4-myc-ROS1-Rep1_S14_L002.bw
ChIPseq-SET4-myc-ROS1xSDG2-ZF-Rep1_S24_L002.bw
ChIPseq-SET5-H2AZ-Col0-Rep2_S33_L003.bw
ChIPseq-SET5-H2AZ-SDG2-ZF-Rep2_S34_L003.bw
ChIPseq-SET5-input-Col0-Rep2_S35_L003.bw
ChIPseq-SET5-input-SDG2-ZF-Rep2_S36_L003.bw
ChIPseq-SET6-H2AZ-SDG2-ZF_rdd-Rep1_S20_L001.bw
ChIPseq-SET6-H2AZ-rdd-Rep1_S19_L001.bw
ChIPseq-SET6-H3K4me3-SDG2-ZF_rdd-Rep1_S18_L001.bw
ChIPseq-SET6-H3K4me3-rdd-Rep1_S17_L001.bw
ChIPseq-SET6-input-Col0_S21_L001.bw
ChIPseq-SET6-input-JMJ14_S25_L001.bw
ChIPseq-SET6-input-JMJ14xTRBIP1-ZF-Rep1_S26_L001.bw
ChIPseq-SET6-input-SDG2-ZF_rdd-Rep1_S28_L001.bw
ChIPseq-SET6-input-rdd-Rep1_S27_L001.bw
ChIPseq-SET6-myc-Col0_S11_L001.bw
ChIPseq-SET6-myc-JMJ14_S15_L001.bw
ChIPseq-SET6-myc-JMJ14xTRBIP1-ZF-Rep1_S16_L001.bw
ChIPseq-SET7-H3K14ac-SDG2-ZF_rdd-Rep2_S50_L003.bw
ChIPseq-SET7-H3K14ac-rdd-Rep2_S49_L003.bw
ChIPseq-SET7-H3K4me3-SDG2-ZF_rdd-Rep2_S46_L003.bw
ChIPseq-SET7-H3K4me3-rdd-Rep2_S45_L003.bw
ChIPseq-SET7-input-SDG2-ZF_rdd-Rep2_S52_L003.bw
ChIPseq-SET7-input-rdd-Rep2_S51_L003.bw
ChIPseq-SET7-myc-Col0-Rep2_S41_L003.bw
ChIPseq-SET7-myc-JMJ14xTRBIP1-ZF-Rep2_S42_L003.bw
ChIPseq-SET7-myc-ROS1-Rep2_S37_L003.bw
ChIPseq-SET7-myc-ROS1xSDG2-ZF-Rep2_S47_L003.bw
ChIPseq-SET8-H3-TRBIP1-ZF-Rep1_S64_L004.bw
ChIPseq-SET8-H3-fwa-Rep1_S63_L004.bw
ChIPseq-SET8-H3K4me3-TRBIP1-ZF-Rep1_S66_L004.bw
ChIPseq-SET8-H3K4me3-fwa-Rep1_S65_L004.bw

ChIPseq-SET9-H3K4me3-TRBIP1-ZF-Rep2-R2_S14_L004.bw
ChIPseq-SET9-H3K4me3-fwa-Rep2-R2_S13_L004.bw
ChIPseq-SET9-input-TRBIP1-ZF-Rep2--R2_S30_L004.bw
ChIPseq-SET9-input-fwa-Rep2-R2_S29_L004.bw
ChIPseq-SET1-H3K4me3-Col0-Rep1_S28_L002_R1_001.fastq.gz
ChIPseq-SET1-H3K4me3-Col0-Rep1_S28_L002_R2_001.fastq.gz
ChIPseq-SET1-H3K4me3-Col0-Rep2_S29_L002_R1_001.fastq.gz
ChIPseq-SET1-H3K4me3-Col0-Rep2_S29_L002_R2_001.fastq.gz
ChIPseq-SET1-H3K4me3-SDG2-ZF-Rep1_S30_L002_R1_001.fastq.gz
ChIPseq-SET1-H3K4me3-SDG2-ZF-Rep1_S30_L002_R2_001.fastq.gz
ChIPseq-SET1-H3K4me3-SDG2-ZF-Rep2_S31_L002_R1_001.fastq.gz
ChIPseq-SET1-H3K4me3-SDG2-ZF-Rep2_S31_L002_R2_001.fastq.gz
ChIPseq-SET1-H3K4me3-SDG2-ZF-Rep3_S32_L002_R1_001.fastq.gz
ChIPseq-SET1-H3K4me3-SDG2-ZF-Rep3_S32_L002_R2_001.fastq.gz
ChIPseq-SET1-input-Col0-Rep1_S43_L002_R1_001.fastq.gz
ChIPseq-SET1-input-Col0-Rep1_S43_L002_R2_001.fastq.gz
ChIPseq-SET1-input-Col0-Rep2_S44_L002_R1_001.fastq.gz
ChIPseq-SET1-input-Col0-Rep2_S44_L002_R2_001.fastq.gz
ChIPseq-SET1-input-SDG2-ZF-Rep1_S45_L002_R1_001.fastq.gz
ChIPseq-SET1-input-SDG2-ZF-Rep1_S45_L002_R2_001.fastq.gz
ChIPseq-SET1-input-SDG2-ZF-Rep2_S46_L002_R1_001.fastq.gz
ChIPseq-SET1-input-SDG2-ZF-Rep2_S46_L002_R2_001.fastq.gz
ChIPseq-SET1-input-SDG2-ZF-Rep3_S47_L002_R1_001.fastq.gz
ChIPseq-SET1-input-SDG2-ZF-Rep3_S47_L002_R2_001.fastq.gz
ChIPseq-SET10-H3K4me3-TRBIP1-dCAS9-MQ1v-Rep1_S22_L004_R1_001.fastq.gz
ChIPseq-SET10-H3K4me3-TRBIP1-dCAS9-MQ1v-Rep1_S22_L004_R2_001.fastq.gz
ChIPseq-SET10-H3K4me3-TRBIP1-dCAS9-MQ1v-Rep2_S26_L004_R1_001.fastq.gz
ChIPseq-SET10-H3K4me3-TRBIP1-dCAS9-MQ1v-Rep2_S26_L004_R2_001.fastq.gz
ChIPseq-SET10-H3K4me3-dCAS9-MQ1v_S23_L004_R1_001.fastq.gz
ChIPseq-SET10-H3K4me3-dCAS9-MQ1v_S23_L004_R2_001.fastq.gz
ChIPseq-SET10-H3K4me3-fwaxrdr6-Rep1_S21_L004_R1_001.fastq.gz
ChIPseq-SET10-H3K4me3-fwaxrdr6-Rep1_S21_L004_R2_001.fastq.gz
ChIPseq-SET10-H3K4me3-fwaxrdr6-Rep2_S25_L004_R1_001.fastq.gz
ChIPseq-SET10-H3K4me3-fwaxrdr6-Rep2_S25_L004_R2_001.fastq.gz
ChIPseq-SET2-H3K4me3-SunTag-SDG2_rdr6-Rep1_S7_L003_R1_001.fastq.gz
ChIPseq-SET2-H3K4me3-SunTag-SDG2_rdr6-Rep1_S7_L003_R2_001.fastq.gz
ChIPseq-SET2-H3K4me3-SunTag-SDG2_rdr6-Rep2_S8_L003_R1_001.fastq.gz
ChIPseq-SET2-H3K4me3-SunTag-SDG2_rdr6-Rep2_S8_L003_R2_001.fastq.gz
ChIPseq-SET2-H3K4me3-noguideSDG2_rdr6_S6_L003_R1_001.fastq.gz
ChIPseq-SET2-H3K4me3-noguideSDG2_rdr6_S6_L003_R2_001.fastq.gz
ChIPseq-SET2-H3K4me3-rdr6-Rep1_S4_L003_R1_001.fastq.gz
ChIPseq-SET2-H3K4me3-rdr6-Rep1_S4_L003_R2_001.fastq.gz
ChIPseq-SET2-H3K4me3-rdr6-Rep2_S5_L003_R1_001.fastq.gz
ChIPseq-SET2-H3K4me3-rdr6-Rep2_S5_L003_R2_001.fastq.gz
ChIPseq-SET2-input-SunTag-SDG2_rdr6-Rep1_S25_L003_R1_001.fastq.gz
ChIPseq-SET2-input-SunTag-SDG2_rdr6-Rep1_S25_L003_R2_001.fastq.gz
ChIPseq-SET2-input-SunTag-SDG2_rdr6-Rep2_S26_L003_R1_001.fastq.gz
ChIPseq-SET2-input-SunTag-SDG2_rdr6-Rep2_S26_L003_R2_001.fastq.gz
ChIPseq-SET2-input-noguideSDG2_rdr6_S24_L003_R1_001.fastq.gz
ChIPseq-SET2-input-noguideSDG2_rdr6_S24_L003_R2_001.fastq.gz
ChIPseq-SET2-input-rdr6-Rep1_S22_L003_R1_001.fastq.gz
ChIPseq-SET2-input-rdr6-Rep1_S22_L003_R2_001.fastq.gz
ChIPseq-SET2-input-rdr6-Rep2_S23_L003_R1_001.fastq.gz
ChIPseq-SET2-input-rdr6-Rep2_S23_L003_R2_001.fastq.gz
ChIPseq-SET3-H3K14ac-Col0-Rep1_S11_L003_R1_001.fastq.gz
ChIPseq-SET3-H3K14ac-Col0-Rep1_S11_L003_R2_001.fastq.gz
ChIPseq-SET3-H3K14ac-Col0-Rep2_S12_L003_R1_001.fastq.gz
ChIPseq-SET3-H3K14ac-Col0-Rep2_S12_L003_R2_001.fastq.gz
ChIPseq-SET3-H3K14ac-SDG2-ZF-Rep1_S15_L003_R1_001.fastq.gz
ChIPseq-SET3-H3K14ac-SDG2-ZF-Rep1_S15_L003_R2_001.fastq.gz
ChIPseq-SET3-H3K14ac-SDG2-ZF-Rep2_S16_L003_R1_001.fastq.gz
ChIPseq-SET3-H3K14ac-SDG2-ZF-Rep2_S16_L003_R2_001.fastq.gz
ChIPseq-SET3-input-Col0-Rep1_S29_L003_R1_001.fastq.gz
ChIPseq-SET3-input-Col0-Rep1_S29_L003_R2_001.fastq.gz
ChIPseq-SET3-input-Col0-Rep2_S30_L003_R1_001.fastq.gz
ChIPseq-SET3-input-Col0-Rep2_S30_L003_R2_001.fastq.gz
ChIPseq-SET3-input-SDG2-ZF-Rep1_S31_L003_R1_001.fastq.gz
ChIPseq-SET3-input-SDG2-ZF-Rep1_S31_L003_R2_001.fastq.gz
ChIPseq-SET3-input-SDG2-ZF-Rep2_S32_L003_R1_001.fastq.gz
ChIPseq-SET3-input-SDG2-ZF-Rep2_S32_L003_R2_001.fastq.gz
ChIPseq-SET4-H2AZ-Col0-Rep1_S20_L002_R1_001.fastq.gz
ChIPseq-SET4-H2AZ-Col0-Rep1_S20_L002_R2_001.fastq.gz
ChIPseq-SET4-H2AZ-SDG2-ZF-Rep1_S21_L002_R1_001.fastq.gz
ChIPseq-SET4-H2AZ-SDG2-ZF-Rep1_S21_L002_R2_001.fastq.gz
ChIPseq-SET4-H2AZ-SDG2-ZF-Rep3_S22_L002_R1_001.fastq.gz
ChIPseq-SET4-H2AZ-SDG2-ZF-Rep3_S22_L002_R2_001.fastq.gz

ChIPseq-SET4-input-Col0-Rep1_S31_L002_R1_001.fastq.gz
ChIPseq-SET4-input-Col0-Rep1_S31_L002_R2_001.fastq.gz
ChIPseq-SET4-input-Col0-Rep2_S32_L002_R1_001.fastq.gz
ChIPseq-SET4-input-Col0-Rep2_S32_L002_R2_001.fastq.gz
ChIPseq-SET4-input-ROS1-Rep1_S26_L002_R1_001.fastq.gz
ChIPseq-SET4-input-ROS1-Rep1_S26_L002_R2_001.fastq.gz
ChIPseq-SET4-input-ROS1xSDG2-ZF-Rep1_S36_L002_R1_001.fastq.gz
ChIPseq-SET4-input-ROS1xSDG2-ZF-Rep1_S36_L002_R2_001.fastq.gz
ChIPseq-SET4-input-SDG2-ZF-Rep1_S33_L002_R1_001.fastq.gz
ChIPseq-SET4-input-SDG2-ZF-Rep1_S33_L002_R2_001.fastq.gz
ChIPseq-SET4-input-SDG2-ZF-Rep3_S34_L002_R1_001.fastq.gz
ChIPseq-SET4-input-SDG2-ZF-Rep3_S34_L002_R2_001.fastq.gz
ChIPseq-SET4-myc-Col0-Rep1_S19_L002_R1_001.fastq.gz
ChIPseq-SET4-myc-Col0-Rep1_S19_L002_R2_001.fastq.gz

ChIPseq-SET4-myc-ROS1-Rep1_S14_L002_R1_001.fastq.gz
ChIPseq-SET4-myc-ROS1-Rep1_S14_L002_R2_001.fastq.gz
ChIPseq-SET4-myc-ROS1xSDG2-ZF-Rep1_S24_L002_R1_001.fastq.gz
ChIPseq-SET4-myc-ROS1xSDG2-ZF-Rep1_S24_L002_R2_001.fastq.gz
ChIPseq-SET5-H2AZ-Col0-Rep2_S33_L003_R1_001.fastq.gz
ChIPseq-SET5-H2AZ-Col0-Rep2_S33_L003_R2_001.fastq.gz
ChIPseq-SET5-H2AZ-SDG2-ZF-Rep2_S34_L003_R1_001.fastq.gz
ChIPseq-SET5-H2AZ-SDG2-ZF-Rep2_S34_L003_R2_001.fastq.gz
ChIPseq-SET5-input-Col0-Rep2_S35_L003_R1_001.fastq.gz
ChIPseq-SET5-input-Col0-Rep2_S35_L003_R2_001.fastq.gz
ChIPseq-SET5-input-SDG2-ZF-Rep2_S36_L003_R1_001.fastq.gz
ChIPseq-SET5-input-SDG2-ZF-Rep2_S36_L003_R2_001.fastq.gz
ChIPseq-SET6-H2AZ-SDG2-ZF_rdd-Rep1_S20_L001_R1_001.fastq.gz
ChIPseq-SET6-H2AZ-SDG2-ZF_rdd-Rep1_S20_L001_R2_001.fastq.gz
ChIPseq-SET6-H2AZ-rdd-Rep1_S19_L001_R1_001.fastq.gz
ChIPseq-SET6-H2AZ-rdd-Rep1_S19_L001_R2_001.fastq.gz
ChIPseq-SET6-H3K4me3-SDG2-ZF_rdd-Rep1_S18_L001_R1_001.fastq.gz
ChIPseq-SET6-H3K4me3-SDG2-ZF_rdd-Rep1_S18_L001_R2_001.fastq.gz
ChIPseq-SET6-H3K4me3-rdd-Rep1_S17_L001_R1_001.fastq.gz
ChIPseq-SET6-H3K4me3-rdd-Rep1_S17_L001_R2_001.fastq.gz
ChIPseq-SET6-input-Col0_S21_L001_R1_001.fastq.gz
ChIPseq-SET6-input-Col0_S21_L001_R2_001.fastq.gz
ChIPseq-SET6-input-JMJ14_S25_L001_R1_001.fastq.gz
ChIPseq-SET6-input-JMJ14_S25_L001_R2_001.fastq.gz
ChIPseq-SET6-input-JMJ14xTRBIP1-ZF-Rep1_S26_L001_R1_001.fastq.gz
ChIPseq-SET6-input-JMJ14xTRBIP1-ZF-Rep1_S26_L001_R2_001.fastq.gz
ChIPseq-SET6-input-SDG2-ZF_rdd-Rep1_S28_L001_R1_001.fastq.gz
ChIPseq-SET6-input-SDG2-ZF_rdd-Rep1_S28_L001_R2_001.fastq.gz
ChIPseq-SET6-input-rdd-Rep1_S27_L001_R1_001.fastq.gz
ChIPseq-SET6-input-rdd-Rep1_S27_L001_R2_001.fastq.gz
ChIPseq-SET6-myc-Col0_S11_L001_R1_001.fastq.gz
ChIPseq-SET6-myc-Col0_S11_L001_R2_001.fastq.gz
ChIPseq-SET6-myc-JMJ14_S15_L001_R1_001.fastq.gz
ChIPseq-SET6-myc-JMJ14_S15_L001_R2_001.fastq.gz
ChIPseq-SET6-myc-JMJ14xTRBIP1-ZF-Rep1_S16_L001_R1_001.fastq.gz
ChIPseq-SET6-myc-JMJ14xTRBIP1-ZF-Rep1_S16_L001_R2_001.fastq.gz
ChIPseq-SET7-H3K14ac-SDG2-ZF_rdd-Rep2_S50_L003_R1_001.fastq.gz
ChIPseq-SET7-H3K14ac-SDG2-ZF_rdd-Rep2_S50_L003_R2_001.fastq.gz
ChIPseq-SET7-H3K14ac-rdd-Rep2_S49_L003_R1_001.fastq.gz
ChIPseq-SET7-H3K14ac-rdd-Rep2_S49_L003_R2_001.fastq.gz
ChIPseq-SET7-H3K4me3-SDG2-ZF_rdd-Rep2_S46_L003_R1_001.fastq.gz
ChIPseq-SET7-H3K4me3-SDG2-ZF_rdd-Rep2_S46_L003_R2_001.fastq.gz
ChIPseq-SET7-H3K4me3-rdd-Rep2_S45_L003_R1_001.fastq.gz
ChIPseq-SET7-H3K4me3-rdd-Rep2_S45_L003_R2_001.fastq.gz
ChIPseq-SET7-input-SDG2-ZF_rdd-Rep2_S52_L003_R1_001.fastq.gz
ChIPseq-SET7-input-SDG2-ZF_rdd-Rep2_S52_L003_R2_001.fastq.gz
ChIPseq-SET7-input-rdd-Rep2_S51_L003_R1_001.fastq.gz
ChIPseq-SET7-input-rdd-Rep2_S51_L003_R2_001.fastq.gz
ChIPseq-SET7-myc-Col0-Rep2_S41_L003_R1_001.fastq.gz
ChIPseq-SET7-myc-Col0-Rep2_S41_L003_R2_001.fastq.gz
ChIPseq-SET7-myc-JMJ14xTRBIP1-ZF-Rep2_S42_L003_R1_001.fastq.gz
ChIPseq-SET7-myc-JMJ14xTRBIP1-ZF-Rep2_S42_L003_R2_001.fastq.gz
ChIPseq-SET7-myc-ROS1-Rep2_S37_L003_R1_001.fastq.gz
ChIPseq-SET7-myc-ROS1-Rep2_S37_L003_R2_001.fastq.gz
ChIPseq-SET7-myc-ROS1xSDG2-ZF-Rep2_S47_L003_R1_001.fastq.gz
ChIPseq-SET7-myc-ROS1xSDG2-ZF-Rep2_S47_L003_R2_001.fastq.gz
ChIPseq-SET8-H3-TRBIP1-ZF-Rep1_S64_L004_R1_001.fastq.gz
ChIPseq-SET8-H3-TRBIP1-ZF-Rep1_S64_L004_R2_001.fastq.gz
ChIPseq-SET8-H3-fwa-Rep1_S63_L004_R1_001.fastq.gz
ChIPseq-SET8-H3-fwa-Rep1_S63_L004_R2_001.fastq.gz
ChIPseq-SET8-H3K4me3-TRBIP1-ZF-Rep1_S66_L004_R1_001.fastq.gz

ChIPseq-SET8-H3K4me3-TRBIP1-ZF-Rep1_S66_L004_R2_001.fastq.gz
ChIPseq-SET8-H3K4me3-fwa-Rep1_S65_L004_R1_001.fastq.gz
ChIPseq-SET8-H3K4me3-fwa-Rep1_S65_L004_R2_001.fastq.gz
ChIPseq-SET9-H3K4me3-TRBIP1-ZF-Rep2-R2_S14_L004_R1_001.fastq.gz
ChIPseq-SET9-H3K4me3-TRBIP1-ZF-Rep2-R2_S14_L004_R2_001.fastq.gz
ChIPseq-SET9-H3K4me3-fwa-Rep2-R2_S13_L004_R1_001.fastq.gz
ChIPseq-SET9-H3K4me3-fwa-Rep2-R2_S13_L004_R2_001.fastq.gz
ChIPseq-SET9-input-TRBIP1-ZF-Rep2--R2_S30_L004_R1_001.fastq.gz
ChIPseq-SET9-input-TRBIP1-ZF-Rep2-R2_S30_L004_R2_001.fastq.gz
ChIPseq-SET9-input-fwa-Rep2-R2_S29_L004_R1_001.fastq.gz
ChIPseq-SET9-input-fwa-Rep2--R2_S29_L004_R2_001.fastq.gz

Genome browser session
(e.g. UCSC)

Available at GEO

Methodology

Replicates

2

Sequencing depth

ChIPseq-SET1-H3K4me3-Col0-Rep1_S28_L002 34936342 19691885 150 PE
ChIPseq-SET1-H3K4me3-Col0-Rep2_S29_L002 38490339 29587724 150 PE
ChIPseq-SET1-H3K4me3-SDG2-ZF-Rep1_S30_L002 47725950 38399503 150 PE
ChIPseq-SET1-H3K4me3-SDG2-ZF-Rep2_S31_L002 36578859 29152566 150 PE
ChIPseq-SET1-H3K4me3-SDG2-ZF-Rep3_S32_L002 50683439 41880615 150 PE
ChIPseq-SET1-input-Col0-Rep1_S43_L002 42857857 35675467 150 PE
ChIPseq-SET1-input-Col0-Rep2_S44_L002 41777637 35657968 150 PE
ChIPseq-SET1-input-SDG2-ZF-Rep1_S45_L002 45414658 39916436 150 PE
ChIPseq-SET1-input-SDG2-ZF-Rep2_S46_L002 38468103 32822196 150 PE
ChIPseq-SET1-input-SDG2-ZF-Rep3_S47_L002 42507063 37646214 150 PE
ChIPseq-SET10-H3K4me3-dCAS9-MQ1v_S23_L004 43443684 20426444 150 PE
ChIPseq-SET10-H3K4me3-fwaxrdr6-Rep1_S21_L004 49828479 25645326 150 PE
ChIPseq-SET10-H3K4me3-fwaxrdr6-Rep2_S25_L004 48373358 24251811 150 PE
ChIPseq-SET10-H3K4me3-TRBIP1-dCAS9-MQ1v-Rep1_S22_L004 51974835 26867737 150 PE
ChIPseq-SET10-H3K4me3-TRBIP1-dCAS9-MQ1v-Rep2_S26_L004 39751228 22853058 150 PE
ChIPseq-SET2-H3K4me3-noguideSDG2_rdr6_S6_L003 28752690 14001658 150 PE
ChIPseq-SET2-H3K4me3-rdr6-Rep1_S4_L003 33757209 17620946 150 PE
ChIPseq-SET2-H3K4me3-rdr6-Rep2_S5_L003 50727153 30571411 150 PE
ChIPseq-SET2-H3K4me3-SunTag-SDG2_rdr6-Rep1_S7_L003 34018551 19227805 150 PE
ChIPseq-SET2-H3K4me3-SunTag-SDG2_rdr6-Rep2_S8_L003 38373436 20107356 150 PE
ChIPseq-SET2-input-noguideSDG2_rdr6_S24_L003 57014877 40285556 150 PE
ChIPseq-SET2-input-rdr6-Rep1_S22_L003 64361592 47134981 150 PE
ChIPseq-SET2-input-rdr6-Rep2_S23_L003 68683440 52026943 150 PE
ChIPseq-SET2-input-SunTag-SDG2_rdr6-Rep1_S25_L003 54532320 40364446 150 PE
ChIPseq-SET2-input-SunTag-SDG2_rdr6-Rep2_S26_L003 68149684 51223468 150 PE
ChIPseq-SET3-H3K14ac-Col0-Rep1_S11_L003 41418247 22473314 150 PE
ChIPseq-SET3-H3K14ac-Col0-Rep2_S12_L003 36908967 19241649 150 PE
ChIPseq-SET3-H3K14ac-SDG2-ZF-Rep1_S15_L003 51202860 28229933 150 PE
ChIPseq-SET3-H3K14ac-SDG2-ZF-Rep2_S16_L003 44728549 24324705 150 PE
ChIPseq-SET3-input-Col0-Rep1_S29_L003 50411344 37539572 150 PE
ChIPseq-SET3-input-Col0-Rep2_S30_L003 61385626 46423104 150 PE
ChIPseq-SET3-input-SDG2-ZF-Rep1_S31_L003 57889292 43964016 150 PE
ChIPseq-SET3-input-SDG2-ZF-Rep2_S32_L003 61370161 47162359 150 PE
ChIPseq-SET4-H2AZ-Col0-Rep1_S20_L002 47629570 27249358 150 PE
ChIPseq-SET4-H2AZ-SDG2-ZF-Rep1_S21_L002 38375021 16374217 150 PE
ChIPseq-SET4-H2AZ-SDG2-ZF-Rep3_S22_L002 30149682 12290991 150 PE
ChIPseq-SET4-input-Col0-Rep1_S31_L002 33273778 28303414 150 PE
ChIPseq-SET4-input-Col0-Rep1_S32_L002 37496935 27549269 150 PE
ChIPseq-SET4-input-ROS1-Rep1_S26_L002 34408865 26412604 150 PE
ChIPseq-SET4-input-ROS1xSDG2-ZF-Rep1_S36_L002 38169834 31807247 150 PE
ChIPseq-SET4-input-SDG2-ZF-Rep3_S34_L002 25826379 17536208 150 PE
ChIPseq-SET4-input-SDG2-ZF-Rep_S33_L002 33609039 23281311 150 PE
ChIPseq-SET4-myc-Col0-Rep1_S19_L002 77486774 34812366 150 PE
ChIPseq-SET4-myc-ROS1-Rep1_S14_L002 30135549 12992580 150 PE
ChIPseq-SET4-myc-ROS1xSDG2-ZF-Rep1_S24_L002 46829138 23023300 150 PE
ChIPseq-SET5-H2AZ-Col0-Rep2_S33_L003 47665823 21462218 150 PE
ChIPseq-SET5-H2AZ-SDG2-ZF-Rep2_S34_L003 53615581 28103163 150 PE
ChIPseq-SET5-input-Col0-Rep2_S35_L003 21480929 13224251 150 PE
ChIPseq-SET5-input-SDG2-ZF-Rep2_S36_L003 42542455 29899785 150 PE
ChIPseq-SET6-H2AZ-rdd-Rep1_S19_L001 11348222 6399582 150 PE
ChIPseq-SET6-H2AZ-SDG2-ZF_rdd-Rep1_S20_L001 17475775 11823941 150 PE
ChIPseq-SET6-H3K4me3-rdd-Rep1_S17_L001 17738930 8282901 150 PE
ChIPseq-SET6-H3K4me3-SDG2-ZF_rdd-Rep1_S18_L001 16672881 10775433 150 PE
ChIPseq-SET6-input-Col0_S21_L001 26717822 23531759 150 PE
ChIPseq-SET6-input-JMJ14_S25_L001 11464571 8153524 150 PE
ChIPseq-SET6-input-JMJ14xTRBIP1-ZF-Rep1_S26_L001 24587909 20064070 150 PE

ChIPseq-SET6-input-rdd-Rep1_S27_L001 22777868 17644762 150 PE
ChIPseq-SET6-input-SDG2-ZF_rdd-Rep1_S28_L001 27442288 22425356 150 PE
ChIPseq-SET6-myc-Col0_S11_L001 46971740 10666018 150 PE
ChIPseq-SET6-myc-JMJ14_S15_L001 24832632 6904089 150 PE
ChIPseq-SET6-myc-JMJ14xTRBIP1-ZF-Rep1_S16_L001 37224404 13829359 150 PE
ChIPseq-SET7-H3K14ac-rdd-Rep2_S49_L003 22359520 9962166 150 PE
ChIPseq-SET7-H3K14ac-SDG2-ZF_rdd-Rep2_S50_L003 58582858 34159191 150 PE
ChIPseq-SET7-H3K4me3-rdd-Rep2_S45_L003 16633914 7279057 150 PE
ChIPseq-SET7-H3K4me3-SDG2-ZF_rdd-Rep2_S46_L003 55641724 31761398 150 PE
ChIPseq-SET7-input-rdd-Rep2_S51_L003 36035182 24628191 150 PE
ChIPseq-SET7-input-SDG2-ZF_rdd-Rep2_S52_L003 85241671 67945330 150 PE
ChIPseq-SET7-myc-Col0-Rep2_S41_L003 74686694 30749305 150 PE
ChIPseq-SET7-myc-JMJ14xTRBIP1-ZF-Rep2_S42_L003 38622007 12072848 150 PE
ChIPseq-SET7-myc-ROS1-Rep2_S37_L003 43663667 21810187 150 PE
ChIPseq-SET7-myc-ROS1xSDG2-ZF-Rep2_S47_L003 56714018 32242760 150 PE
ChIPseq-SET8-H3-fwa-Rep1_S63_L004 30513471 28253253 150 PE
ChIPseq-SET8-H3-TRBIP1-ZF-Rep1_S64_L004 27295347 24902427 150 PE
ChIPseq-SET8-H3K4me3-fwa-Rep1_S65_L004 32065146 27900637 150 PE
ChIPseq-SET8-H3K4me3-TRBIP1-ZF-Rep1_S66_L004 27753518 22487628 150 PE
ChIPseq-SET9-H3K4me3-fwa-Rep2-R2_S13_L004 17898069 13691730 150 PE
ChIPseq-SET9-H3K4me3-TRBIP1-ZF-Rep2-R2_S14_L004 16739918 11612468 150 PE
ChIPseq-SET9-input-fwa-Rep2-R2_S29_L004 40053656 33839630 150 PE
ChIPseq-SET9-input-TRBIP1-ZF-Rep2--R2_S30_L004 46478573 37721657 150 PE

Antibodies

Anti-Histone H3 Abcam Cat# ab1791, RRID:AB_302613
Anti-trimethyl-Histone H3 (Lys4)¬ Millipore Sigma Cat# 04-745, RRID:AB_1163444
Anti-trimethyl-Histone H3 (Lys27) Millipore Sigma Cat# 07-449, RRID:AB_310624
Anti-Myc-tag (71D10) rabbit mAb Cell Signaling Cat# 2278S, RRID:AB_1658203

Peak calling parameters

MACS2: '-f BAM -g 1.3e+8 -q 0.05 --extsize 147'

Data quality

All identified peaks in the study were called with a qval threshold of 0.01 (FDR 1%).

Software

Bowtie2 (v2.1.0),
Samtools (v1.9)
MACS2 (v2.1.1)
deeptools (v2.5.1).
bedtools (v2.26.0)
Rstudio version 4.1.3
