# Supplementary figures and images for: Histone H3 lysine 4 methylation recruits DNA demethylases to enforce gene expression in Arabidopsis
Source: Nat Plants. 2025 Feb 11;11(2):206–17. doi: 10.1038/s41477-025-01924-y (PMC11842272; doi:10.1038/s41477-025-01924-y)

Source data of Extended Data Fig. 1a

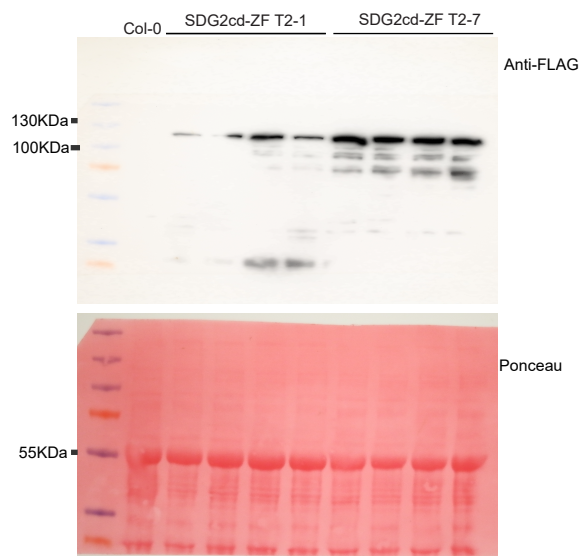

Supplement: Supplementary file 4 — Unprocessed western blots for Extended Data Fig. 1a. [file 41477_2025_1924_MOESM4_ESM.pdf]

Extended Data Fig. 2b

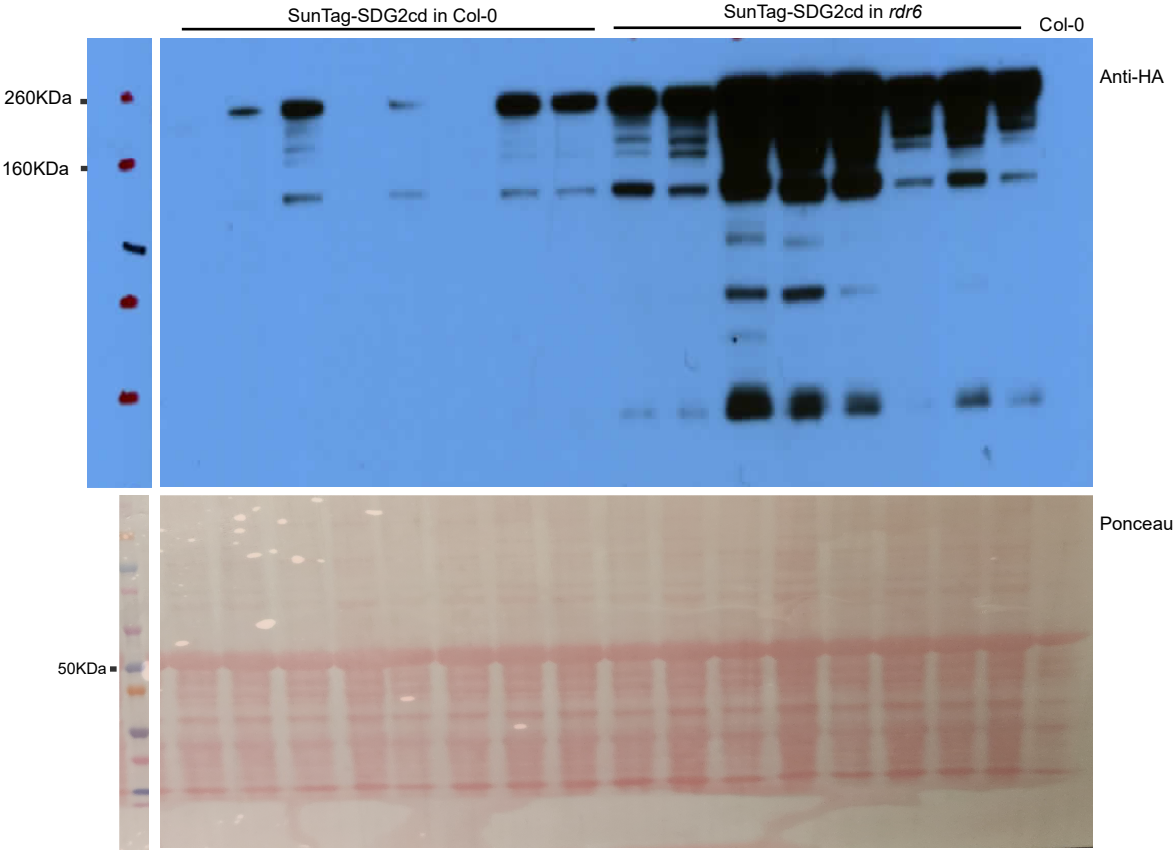

Supplement: Supplementary file 5 — Unprocessed western blots for Extended Data Fig. 2b. [file 41477_2025_1924_MOESM5_ESM.pdf]

Source data of Extended Data Fig. 7e

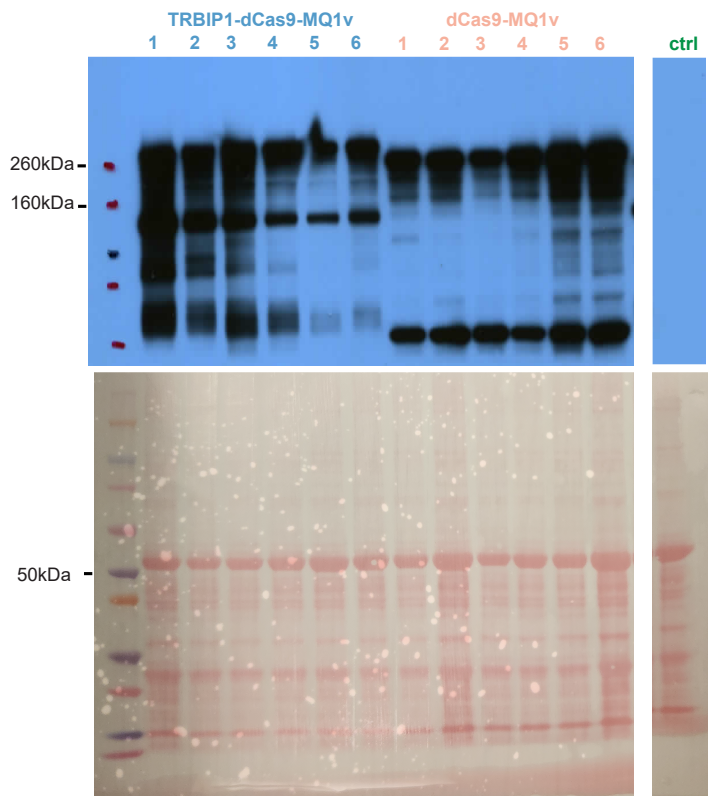

Supplement: Supplementary file 7 — Unprocessed western blots for Extended Data Fig. 7e. [file 41477_2025_1924_MOESM7_ESM.pdf]
