# Supplementary material for: Histone H3 lysine 4 methylation recruits DNA demethylases to enforce gene expression in Arabidopsis
Source: Nat Plants. 2025 Feb 11;11(2):206–17. doi: 10.1038/s41477-025-01924-y (PMC11842272; doi:10.1038/s41477-025-01924-y)

Source Data File-Extended Data Fig.4

Source Data File-Extended Data Fig.4a up panel

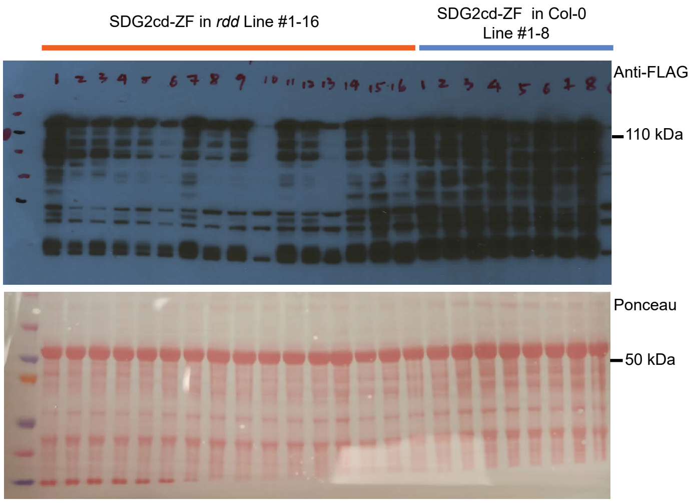

Source Data File-Extended Data Fig.4b

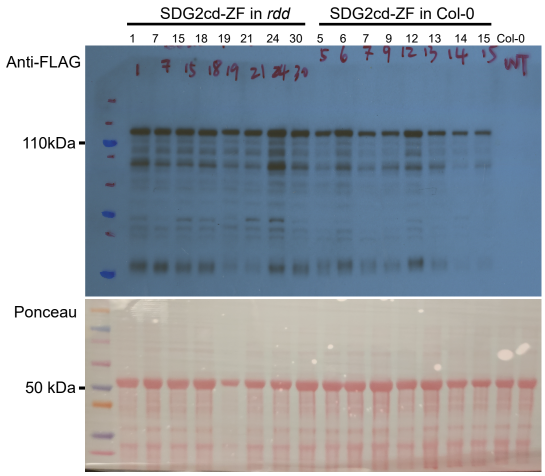

Source Data File-Extended Data Fig.4a bottom panel

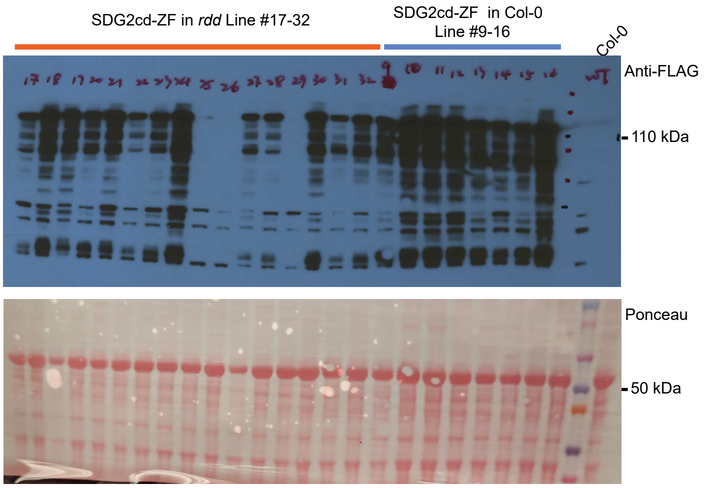

Supplement: Supplementary file 6 — Unprocessed western blots for Extended Data Fig. 4a,b. [file 41477_2025_1924_MOESM6_ESM.pdf]
